# Examining reproducibility in psychology: A hybrid method for combining a statistically significant original study and a replication

**DOI:** 10.3758/s13428-017-0967-6

**Published:** 2017-09-21

**Authors:** Robbie C. M. van Aert, Marcel A. L. M. van Assen

**Affiliations:** 1 Department of Methodology and Statistics, Tilburg University, P.O. Box 90153, 5000 LE Tilburg, The Netherlands; 2 Department of Sociology, Utrecht University, Utrecht, The Netherlands

**Keywords:** Replication, Meta-analysis, *p*-Uniform, Reproducibility

## Abstract

The unrealistically high rate of positive results within psychology has increased the attention to replication research. However, researchers who conduct a replication and want to statistically combine the results of their replication with a statistically significant original study encounter problems when using traditional meta-analysis techniques. The original study’s effect size is most probably overestimated because it is statistically significant, and this bias is not taken into consideration in traditional meta-analysis. We have developed a hybrid method that does take the statistical significance of an original study into account and enables (a) accurate effect size estimation, (b) estimation of a confidence interval, and (c) testing of the null hypothesis of no effect. We analytically approximate the performance of the hybrid method and describe its statistical properties. By applying the hybrid method to data from the Reproducibility Project: Psychology (Open Science Collaboration, 2015), we demonstrate that the conclusions based on the hybrid method are often in line with those of the replication, suggesting that many published psychological studies have smaller effect sizes than those reported in the original study, and that some effects may even be absent. We offer hands-on guidelines for how to statistically combine an original study and replication, and have developed a Web-based application (https://rvanaert.shinyapps.io/hybrid) for applying the hybrid method.

**Electronic supplementary material:**

The online version of this article (10.3758/s13428-017-0967-6) contains supplementary material, which is available to authorized users.

Increased attention is being paid to replication research in psychology, mainly due to the unrealistic high rate of positive results within the published psychological literature. Approximately 95% of the published psychological research contains statistically significant results in the predicted direction (Fanelli, 2012; Sterling, Rosenbaum, & Weinkam, 1995). This is not in line with the average amount of statistical power, which has been estimated at .35 (Bakker, van Dijk, & Wicherts, 2012) or .47 (Cohen, 1990) in psychological research and .21 in neuroscience (Button et al., 2013), indicating that statistically nonsignificant results often do not get published. This suppression of statistically nonsignificant results from being published is called publication bias (Rothstein, Sutton, & Borenstein, 2005). Publication bias causes the population effect size to be overestimated (e.g., Lane & Dunlap, 1978; van Assen, van Aert, & Wicherts, 2015) and raises the question whether a particular effect reported in the literature actually exists. Other research fields have also shown an excess of positive results (e.g., Ioannidis, 2011; Kavvoura et al., 2008; Renkewitz, Fuchs, & Fiedler, 2011; Tsilidis, Papatheodorou, Evangelou, & Ioannidis, 2012), so publication bias and the overestimation of effect size by published research is not only an issue within psychology.

Replication research can help to identify whether a particular effect in the literature is probably a false positive (Murayama, Pekrun, & Fiedler, 2014), and to increase accuracy and precision of effect size estimation. The Open Science Collaboration carried out a large-scale replication study to examine the reproducibility of psychological research (Open Science Collaboration, 2015). In this so-called Reproducibility Project: Psychology (RPP), articles were sampled from the 2008 issues of three prominent and high-impact psychology journals and a key effect of each article was replicated according to a structured protocol. The results of the replications were not in line with the results of the original studies for the majority of replicated effects. For instance, 97% of the original studies reported a statistically significant effect for a key hypothesis, whereas only 36% of the replicated effects were statistically significant (Open Science Collaboration, 2015). Moreover, the average effect size of the replication studies was substantially smaller (*r* = .197) than those of original studies (*r* = .403). Hence, the results of the RPP confirm both the excess of significant findings and overestimation of published effects within psychology.

The larger effect size estimates in the original studies than in their replications can be explained by the expected value of a statistically significant original study being larger than the true mean (i.e., overestimation). The observed effect size of a replication, which has not (yet) been subjected to selection for statistical significance, will usually be smaller. This statistical principle of an extreme score on a variable (in this case a statistically significant effect size) being followed by a score closer to the true mean is also known as regression to the mean (e.g., Straits & Singleton, 2011, chap. 5). Regression to the mean occurs if simultaneously (i) selection occurs on the first measure (in our case, only statistically significant effects), and (ii) both of the measures are subject to error (in our case, sampling error).

It is crucial to realize that the expected value of statistically significant observed effects of the original studies will be larger than the true effect size *irrespective of the presence of publication bias*. That is, conditional on being statistically significant, the expected value of the original effect size will be larger than the true effect size. The distribution of the statistically significant original effect size is actually a truncated distribution at the critical value, and these effect sizes are larger than the nonsignificant observed effects. Hence, the truncated distribution of statistically significant effects has a larger expected value than the true effect size. Publication bias only determines how often statistically nonsignificant effects get published, and therefore it does not influence the expected value of the statistically significant effects. Consequently, statistical analyses based on an effect that was selected for replication because of its significance should correct for the overestimation in effect size irrespective of the presence of publication bias.

Estimating effect size and determining whether an effect truly does exist on the basis of an original published study and a replication is important. This is not only relevant for projects such as the RPP. Because replicating published research is often the starting point for new research in which the replication is the first study of a multistudy article (Neuliep & Crandall, 1993), it is also relevant for researchers who carry out a replication and want to aggregate the results of the original study and their own replication. Cumming (2012, p. 184) emphasized that combining two studies by means of a meta-analysis has added value over interpreting two studies in isolation. Moreover, researchers in the field of psychology have also started to use meta-analysis to combine the studies within a single article, in what is called an *internal* meta-analysis (Ueno, Fastrich, & Murayama, 2016). Additionally, the proportion of published replication studies will increase in the near future due to the widespread attention to the replicability of psychological research nowadays. Finally, we must note that the Makel, Plucker, and Hegarty’s (2012) estimate of 1% of published studies in psychology being replications is a gross underestimation. They merely searched for the word “replication” and variants thereof in psychological articles. However, researchers often do not label studies as replications, to increase the likelihood of publication (Neuliep & Crandall, 1993), even though many of them carry out a replication before starting their own variation of the study. To conclude, making sense of and combining the results of an original study and a replication is a common and important problem.

The main difficulty with combining an original study and a replication is *how* to aggregate a likely overestimated effect size in the published original study with the unpublished and probably unbiased replication. For instance, what should a researcher conclude when the original study is statistically significant and the replication is not? This situation often arises—for example, of the 100 effects examined in the RPP, in 62% of the cases the original study was statistically significant, whereas the replication was not. To examine the main problem in more detail, consider the following hypothetical situation. Both the original study and replication consist of two independent groups of equal size, with the total sample size in the replication being twice as large as in the original study (80 vs. 160). The researcher may encounter the following standardized effect sizes (Hedges’ *g*),^1^ *t* values, and two-tailed *p* values: *g* = 0.490, *t*(78) = 2.211, *p* = .03, for the original study, and *g =* 0.164, *t*(158) = 1.040, *p* = .3, for the replication. A logical next step for interpreting these results would be to combine the observed effect sizes of both the original study and replication by means of a fixed-effect meta-analysis. The results of such a meta-analysis suggest that there is indeed an effect in the population after combining the studies with meta-analytic effect size estimate $\widehat{\theta}$= 0.270, *z* = 2.081, *p* = .0375 (two-tailed). However, the researcher may not be convinced that the effect really exists and does not know how to proceed, since the original study is probably biased, and the meta-analysis does not take this bias into account.

The aim of this article is threefold. First, we developed a method (i.e., the hybrid method of meta-analysis, *hybrid* for short) that combines a statistically significant original study and replication and that does correct for the likely overestimation in the original study’s effect size estimate. The hybrid method yields (a) an accurate estimate of the underlying population effect based on the original study and the replication, (b) a confidence interval around this effect size estimate, and (c) a test of the null hypothesis of no effect for the combination of the original study and replication. Second, we applied the hybrid and traditional meta-analysis methods to the data of the RPP to examine the reproducibility of psychological research. Third, to assist practical researchers in assessing effect size using an original and replication study, we have formulated guidelines for which method to use under what conditions, and we explain a newly developed Web-based application for estimation based on these methods.

The remainder of the article is structured as follows. We explain traditional meta-analysis and propose the new hybrid method for combining an original study and a replication while taking into account statistical significance of the original study’s effect. We adopt a combination of the frameworks of Fisher and Neyman–Pearson that is nowadays commonly used in practice to develop and examine our procedures for testing and estimating effect size. Next, we analytically approximate the performance of meta-analysis and the hybrid method in a situation in which an original study and its replication are combined. The performance of meta-analysis and the hybrid method are compared to each other, and to estimation using only the replication. On the basis of the performance of the methods, we formulate guidelines on which method to use under what conditions. Subsequently, we describe the RPP and apply meta-analysis and the hybrid method to these data. The article concludes with a discussion and an illustration of a Web-based application (https://rvanaert.shinyapps.io/hybrid) allowing straightforward application of the hybrid method to researchers’ applications.

## Methods for estimating effect size

The statistical technique for estimating effect size based on multiple studies is meta-analysis (Borenstein, Hedges, Higgins, & Rothstein, 2009, Preface). The advantage of meta-analysis over interpreting the studies in isolation is that the effect size estimate in a meta-analysis is more precise. Two meta-analysis methods are often used: fixed-effect meta-analysis and random-effects meta-analysis. *Fixed-effect* meta-analysis assumes that one common population effect size underlies the studies in the meta-analysis, whereas *random-effects* meta-analysis assumes that the each study has its own population effect size. The studies’ population effect sizes in random-effects meta-analysis are assumed to be a random sample from a normal distribution of population effect sizes, and one of the aims of random-effects meta-analysis is to estimate the mean of this distribution (e.g., Borenstein et al., 2009, chap. 10). Fixed-effect rather than random-effects meta-analysis is the recommended method to aggregate the findings of an original study and an exact or direct replication, assuming that both studies assess the same underlying population effect. Note also that statistically combining two studies by means of random-effects meta-analysis is practically infeasible, since the amount of heterogeneity among a small number of studies cannot be accurately estimated (e.g., Borenstein, Hedges, Higgins, & Rothstein, 2010; IntHout, Ioannidis, & Borm, 2014). After discussing fixed-effect meta-analysis, we introduce the hybrid method as an alternative method that takes into account the statistical significance of the original study.

## Fixed-effect meta-analysis

Before the average effect size with a meta-analysis can be computed, studies’ effect sizes and sampling variances have to be transformed to one common effect size measure (see Borenstein, 2009; Fleiss & Berlin, 2009). The true effect size (*θ*) is estimated in each study with sampling error (*ε*_i_). This model can be written as

$$ {y}_i=\theta +{\varepsilon}_i, $$

where *y*_i_ reflects the effect size in the *i*th study and it is assumed that the *ε*_i_ is normally and independently distributed, *ε*_i_ ~ *N*(0, ${\sigma}_i^2$) with ${\sigma}_i^2$ being the sampling variance in the population for each study. These sampling variances are assumed to be known in meta-analysis.

The average effect size is computed by weighting each *y*_i_ with the reciprocal of the estimated sampling variance (${w}_i=\frac{1}{{\widehat{\sigma}}_i^2}$). For *k* studies in a meta-analysis, the weighted average effect size estimate ($\widehat{\theta}$) is computed by

1 $$ \widehat{\theta}=\frac{\sum_{i=1}^k{w}_i{y}_i}{\sum_{i=1}^k{w}_i}, $$

with variance

$$ {v}_{\widehat{\theta}}=\frac{1}{\sum_{i=1}^k{w}_i}. $$

A 95% confidence interval around $\widehat{\theta}$ can be obtained by $\widehat{\theta}\pm 1.96\sqrt{v_{\widehat{\theta}}}$ with 1.96 being the 97.5th percentile of the normal distribution and a *z* test can be used to test H_0_: *θ* = 0,

$$ z=\frac{\widehat{\theta}}{\sqrt{v_{\widehat{\theta}}}}. $$

Applying fixed-effect meta-analysis to the example as presented in the introduction, we first have to compute the sampling variance of the Hedges’ *g* effect size estimates for the original study and replication. An unbiased estimator of the variance of *y* is computed by

$$ {\widehat{\sigma}}^2=\frac{1}{n_1}+\frac{1}{n_2}+\left[\frac{1-\left({n}_1+{n}_2-4\right)}{\left({n}_1+{n}_2-2\right){J}^2}\right]{g}^2 $$

where *n*_1_ and *n*_2_ are the sample sizes for Groups 1 and 2 (Viechtbauer, 2007). This yields weights 19.390 and 39.863 for the original study and replication, respectively. Computing the fixed-effect meta-analytic estimate (Eq. 1) with *y*_i_ being the Hedges’ *g* observed effect size estimates gives

$$ \widehat{\theta}=\frac{19.390\times 0.490+39.863\times 0.164}{19.390+39.863}=0.270, $$

with the corresponding variance

$$ {v}_{\widehat{\theta}}=\frac{1}{\left(19.390+39.863\right)}=0.017. $$

The 95% confidence interval of the fixed-effect meta-analytic estimate ranges from 0.016 to 0.525, and the null hypothesis of no effect is rejected (*z* = 2.081, two-tailed *p* value = .0375). Note that the *t* distribution was used as reference distribution for testing the original study and replication individually whereas a normal distribution was used in the fixed-effect meta-analysis. The use of a normal distribution as reference distribution in fixed-effect meta-analysis is a consequence of the common assumptions in meta-analysis of known sampling variances and normal sampling distributions of effect size (Raudenbush, 2009).

## Hybrid method

Like fixed-effect meta-analysis, the hybrid method estimates the common effect size of an original study and replication. By taking into account that the original study is statistically significant, the proposed hybrid method corrects for the likely overestimation in the effect size of the original study. The hybrid method is based on the statistical principle that the distribution of *p* values at the true effect size is uniform. A special case of this statistical principle is that the *p* values are uniformly distributed under the null hypothesis (e.g., Hung, O’Neill, Bauer, & Köhne, 1997). This principle also underlies the recently developed meta-analytic techniques *p-*uniform (van Aert, Wicherts, & van Assen, 2016; van Assen et al., 2015) and *p*-curve (Simonsohn, Nelson, & Simmons, 2014a, b). These methods discard statistically nonsignificant effect sizes, and only use the statistically significant effect sizes in a meta-analysis to examine publication bias. *P-*uniform and *p-*curve correct for publication bias by computing probabilities of observing a study’s effect size conditional on the effect size being statistically significant. The effect size estimate of *p-*uniform and *p-*curve equals that effect size for which the distribution of these conditional probabilities is best approximated by a uniform distribution. Both methods yield accurate effect size estimates in the presence of publication bias if heterogeneity in true effect size is at most moderate (Simonsohn et al., 2014a; van Aert et al., 2016, 2015). In contrast to *p*-uniform and *p*-curve, which assume that all included studies are statistically significant, only the original study is assumed to be statistically significant in the hybrid method. This assumption hardly restricts the applicability of the hybrid method since approximately 95% of the published psychological research contains statistically significant results (Fanelli, 2012; Sterling et al., 1995).

To deal with bias in the original study, its *p* value is transformed by computing the probability of observing the effect size or larger conditional on the effect size being statistically significant and at the population effect size (*θ*).^2^ This can be written as

2 $$ {q}_O=\frac{P\left(y\ge {y}_O;\theta \right)}{P\left(y\ge {y}_O^{CV};\theta \right)}, $$

where the numerator refers to the probability of observing a larger effect size than in the original study (*y*_*O*_) at effect size *θ*, and the denominator denotes the probability of observing an effect size larger than its critical value (${y}_O^{CV}$) at effect size *θ*. Note that ${y}_O^{CV}$ is independent of *θ*. The conditional probability *q*_O_ at true effect size *θ* is uniform whenever *y*_O_ is larger than ${y}_O^{CV}$. These conditional probabilities are also used in *p-*uniform for estimation and testing for an effect while correcting for publication bias (van Aert et al., 2016, 2015). The replication is not assumed to be statistically significant, so we compute the probability of observing a larger effect size than in the replication (*q*_R_) at effect size *θ*

3 $$ {q}_R=P\left(y\ge {y}_R;\theta \right), $$

with the observed effect size of the replication denoted by *y*_R_. Both *q*_O_ and *q*_R_ are calculated under the assumption that the sampling distributions of *y*_O_ and *y*_R_ are normally distributed, which is the common assumption in meta-analysis (Raudenbush, 2009).

Testing of H_0_: *θ* = 0 and estimation is based on the principle that each (conditional) probability is uniformly distributed at the true value *θ*. Different methods exist for testing whether a distribution deviates from a uniform distribution. The hybrid method uses the distribution of the sum of independently uniformly distributed random variables (i.e., the Irwin–Hall distribution),^3^ *x* = *q*_O_ + *q*_R_, because this method is intuitive, showed good statistical properties in the context of *p-*uniform, and can also be used for estimating a confidence interval (van Aert et al., 2016). The probability density function of the Irwin–Hall distribution for *x* based on two studies is

$$ f(x)=\left\{\begin{array}{ll}x\hfill & 0\le x\le 1\hfill \\ {}2-x\hfill & 1\le x\le 2\hfill \end{array},\right. $$

and its cumulative distribution function is

4 $$ F(x)=\left\{\begin{array}{cc}\hfill \frac{1}{2}{x}^2\hfill & \hfill 0\le x\le 1\hfill \\ {}\hfill -\frac{1}{2}{x}^2+2x-1\hfill & \hfill 1\le x\le 2\hfill \end{array}\right.. $$

Two-tailed *p* values of the hybrid method can be obtained with *G*(*x*),

5 $$ G(x)=\left\{\begin{array}{cc}\hfill {x}^2\hfill & \hfill 0\le x\le 1\hfill \\ {}\hfill 2-\left(-{x}^2+4x-2\right)\hfill & \hfill 1\le x\le 2\hfill \end{array}\right.. $$

The null hypothesis H_0_: *θ* = 0 is rejected if *F*(*x* | *θ* = 0) ≤ .05 in case of a one-tailed test, and *G*(*x* |*θ* = 0) ≤ .05 in case of a two-tailed test. The 2.5th and 5th percentiles of the Irwin–Hall distribution are 0.224 and 0.316, respectively. Effect size *θ* is estimated as *F*(*x* | *θ* = $\widehat{\theta}$) = .5, or equivalently, that value of *θ* for which *x* = 1. The 95% confidence interval of *θ*, (${\widehat{\theta}}_L,{\widehat{\theta}}_H$), is calculated as *F*(*x* | *θ* = ${\widehat{\theta}}_L$) = .975 and *F*(*x* | *θ* = ${\widehat{\theta}}_H$) = .025.

**Fig. 1:**
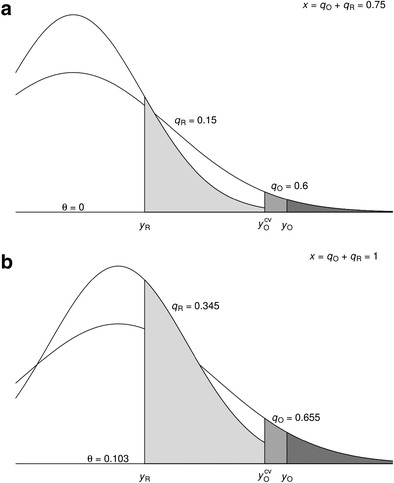
Effect size distributions of the original study and replication for the example presented in the introduction. Panels a and b refer to the effect size distributions for *θ* = 0 and *θ* = 0.103. *y*_O_ and *y*_R_ denote the observed effect sizes in the original study and replication, and ${y}_O^{CV}$ denotes the critical value of the original study based on a two-tailed hypothesis test of H_0_: *θ* = 0 with *α* = .05. The shaded regions refer to probabilities larger than *y*_R_, *y*_O_, and ${y}_O^{CV}$. The (conditional) probabilities of the original study and replication are indicated by *q*_O_ and *q*_R_, and their sum by *x*

We will now apply the hybrid method to the example presented in the introduction. The effect size measure of the example in the introduction is Hedges’ *g*, but the hybrid method can also be applied to an original study and replication in which another effect size measure (e.g., the correlation coefficient) is computed. Figure 1 illustrates the computation of *q*_O_ and *q*_R_ for *θ* = 0 (Fig. 1a) and for *θ* = $\widehat{\theta}$ (Fig. 1b), based on the example presented in the introduction. The steepest distribution in both panels refers to the effect size distribution of the replication, which has the largest sample size. The conditional probability *q*_O_ for *θ* = 0 (Fig. 1a) equals the area larger than ${y}_O^{CV}$ (intermediate gray color) divided by the area larger than *y*_O_ (dark gray): ${q}_O=\frac{0.015}{0.025}=0.6$. The probability *q*_R_ equals the one-tailed *p* value (.3/2 = .15) and is indicated by the light gray area.^4^ Summing these two probabilities gives *x* = .75, which is lower than the expected value of the Irwin–Hall distribution, suggesting that the effect size exceeds 0. The null hypothesis of no effect is not rejected, with a two-tailed *p* value equal to .558 as calculated by Eq. 5. Shifting *θ* to hybrid’s estimate = 0.103 yields *x* = 1, as depicted in Fig. 1b, with *q*_O_ = .655 and *q*_R_ = .345. Estimates of the lower and upper bounds of a 95% confidence interval can also be obtained by shifting $\widehat{\theta}$ until *x* equals the 2.5th and 97.5th percentiles, for the lower and upper bounds of the confidence interval. The confidence interval of the hybrid method for the example ranges from – 1.109 to 0.428.

The results of applying fixed-effect meta-analysis and the hybrid method to the example are summarized in Table 1. The original study suggests that the effect size is medium and statistically significantly different from zero (first row), but the effect size in the replication is small at best and not statistically significant (second row). Fixed-effect meta-analysis (third row) is usually seen as the best estimator of the true effect size in the population and suggests that the effect size is small to medium (0.270) and statistically significant (*p* = .0375). However, the hybrid’s estimate is small (0.103) and not statistically significant (*p* = *.*558) (fourth row). Hybrid’s estimate is lower than the estimate of fixed-effect meta-analysis because it corrects for the first study being statistically significant. Hybrid’s estimate is even lower than the estimate of the replication because, when taking the significance of the original study into account, the original study suggests a zero or even negative effect, which pulls the estimate to zero.

**Table 1:** Effect size estimates (Hedges’*g*), 95% confidence intervals (CI), and two-tailed *p* values of the original study and replication in the hypothetical situation, and results of the fixed-effect meta-analysis and the hybrid, hybrid^0^, and hybrid^R^ methods when applied to the hypothetical situation

Method	$\widehat{\theta}$ (95% CI) [*p* Value]
Original study (*y*_O_)	0.490 (0.044; 0.935) [0.0311]
Replication (*y*_R_)	0.164 (– 0.147; 0.474) [0.302]
Fixed-effect meta-analysis	0.270 (0.016; 0.525) [0.0375]
Hybrid	0.103 (– 1.109; 0.428) [0.558]
Hybrid^0^	0.103 (– 1.109; 0.429) [0.558]
Hybrid^R^	0.164 (– 0.147; 0.474) [0.302]

Van Aert et al. (2016) showed that not only the lower bound of a 95% confidence interval, but also the estimated effect sizes by *p*-uniform can become highly negative if the effect size is estimated on the basis of a single study and its *p* value is close to the alpha level.^5^ The effect size estimates can be highly negative because conditional probabilities such as *q*_O_ are not sensitive to changes in *θ* when the (unconditional) *p* value is close to alpha. Applying *p*-uniform to a single study in which a one-tailed test is conducted with *α* = .05 yields an effect size estimate of *p-*uniform equal to zero if the *p* value is .025, a positive estimate if the *p* value is smaller than .025, a negative estimate if the *p* value is larger than .025, and a highly negative estimate if the *p* value is close to .05. Van Aert et al. (2016) recommended setting the effect size estimate equal to zero if the mean of the primary studies’ *p* values is larger than half the α- level, because *p*-uniform’s effect size estimate will then be below zero. Setting the effect size to 0 is analogous to testing a one-tailed null hypothesis in which the observed effect size is in the opposite direction from the one expected. Computing a test statistic and *p* value is redundant in such a situation, because the test statistic will be negative and the one-tailed *p* value will be above .5.

The hybrid method can also yield highly negative effect size estimates because, like *p*-uniform, it uses a conditional probability for the original study’s effect size. In line with the proposal in van Aert et al. (2016), we developed two alternative hybrid methods, hybrid^0^ and hybrid^R^, to avoid highly negative estimates. The hybrid^0^ method is a direct application of the *p*-uniform method as recommended by van Aert et al., which recommends setting the effect size estimate to 0 if the studies’ combined evidence points to a negative effect. Applied to the hybrid^0^ method, this translates to setting the effect size equal to 0 if *x* > 1 under the null hypothesis, and equal to that of hybrid otherwise. Consequently, hybrid^0^ will, in contrast to hybrid, never yield an effect size estimate that is below zero. Applied to the example, hybrid^0^ equals hybrid’s estimate because *x* = 0.75 under the null hypothesis.

The other alternative hybrid method, hybrid^R^ (where the R refers to *replication*), addresses the problem of highly negative estimates in a different way. The estimate of hybrid^R^ is equal to hybrid’s estimate if the original study’s two-tailed *p* value is smaller than .025 and is equal to the effect size estimate of the replication if the original study’s two-tailed *p* value is larger than .025. A two-tailed *p* value of .025 in the original study is used because this results in a negative effect size estimate, which is not in line with either the theoretical expectation or the observed effect size in the original study. Hence, if the original study’s just statistically significant effect size (i.e., .025 < *p* < .05) points to a negative effect, the evidence of the original study is discarded and only the results of the replication are interpreted. The estimate of hybrid^R^ (and also of hybrid) is not restricted to be in the same direction as the original study as is the case for hybrid^0^. The results of applying hybrid^R^ to the example are presented in the last row of Table 1. Hybrid^R^ only uses the observed effect size in the replication—because the *p* value in the original study, .03, exceeds .025—and hence yields the same results as the replication study, as is reported in the second row.

Since all of the discussed methods may yield different results, it is important to examine their statistical properties. The next section describes the performance of the methods evaluated using an analytical approximation of these methods’ results.

## Performance of estimation methods: Analytical comparison

### Method

We used the correlation coefficient as effect size measure because our application discussed later, the RPP, also used correlations. However, all methods can also deal with other effect size measures as for instance standardized mean differences. We analytically compared the performance of five methods; fixed-effect meta-analysis, estimation using only the replication (maximum likelihood), and the hybrid, hybrid^0^, and hybrid^R^ methods.

We evaluated the methods’ statistical properties by using a procedure analogous to the procedure described in van Aert and van Assen (2017). The methods were applied to the joint probability density function (pdf) of statistically significant original effect size and replication effect size. This joint pdf was a combination of the marginal pdfs of the statistically significant original effect size and the replication effect size, and was approximated by using numerical integration. Both marginal pdfs depended on the true effect size and the sample size in the original study and replication. The marginal pdf of statistically significant original effect sizes was approximated by first creating 1,000 evenly distributed cumulative probabilities or percentiles ${P}_i^O$ of this distribution given true effect size and sample size in the original study, with

$$ {P}_i^O=1-\pi +\frac{\left(i\times \pi \right)}{1,001}. $$

Here, *π* denotes the power of the null hypothesis test of no effect—that is, the probability that effect size exceeds the critical value. We used the Fisher *z* test, with *α* = .025 corresponding to common practice in psychological research in which two-tailed hypothesis tests are conducted and only results in the predicted direction get published. For instance, if the null hypothesis is true the cumulative probabilities ${P}_i^O$ are evenly distributed and range from $1-0.025+\frac{\left(1\times .025\right)}{1,001}=0.975025$ to $1-0.025+\frac{\left(1,000\times .025\right)}{1,001}=0.999975$. Finally, the 1,000 ${P}_i^O$ values were converted by using a normal distribution to the corresponding 1,000 (statistically significant) Fisher-transformed correlation coefficients.

The marginal pdf of the replication was approximated by selecting another 1,000 equally spaced cumulative probabilities given true effect size and sample size of the replication with ${P}_i^R=\frac{i}{1,001}$. These cumulative probabilities range from $\frac{1}{1,001}=0.000999001$ to $\frac{1,000}{1,001}=0.999001$, and were subsequently also transformed to Fisher-transformed correlation coefficients by using a normal distribution. The joint pdf was obtained by multiplying the two statistically independent marginal pdfs, and yielded 1,000×1,000 = 1,000,000 different combinations of statistically significant original effect size and replication effect size. The methods were applied to each of the combination of effect sizes in the original study and replication. For presenting the results, Fisher-transformed correlations were transformed to correlations.^6^

Statistical properties of the different methods were evaluated on the basis of average effect size estimate, median effect size estimate, standard deviation of effect size estimate, root mean square error (RMSE), coverage probability (i.e., the proportion describing how often the true effect size falls inside the confidence interval), and statistical power and Type I error for testing the null hypothesis of no effect. Population effect size (*ρ*) and sample size in the original study (*N*_O_) and replication (*N*_R_) were varied. Values for *ρ* were chosen to reflect no (0), small (0.1), medium (0.3), and large (0.5) true effects, as specified by Cohen (1988, chap. 3). Representative sample sizes within psychology were used for the computations by selecting the first quartile, median, and third quartile of the original study’s sample size in the RPP: 31, 55, and 96. These sample sizes were used for the original study and replication. A sample size of 783 was also included for the replication to reflect a recommended practice in which the sample size is determined with a power analysis to detect a small true effect with a statistical power of 0.8. The computations were conducted in R, using the parallel package for parallel computing (R Development Core Team, 2015). The root-finding bisection method (Adams & Essex, 2013, pp. 85–86) was used to estimate the effect size and the confidence interval of the hybrid method. R code of the analyses is available via https://osf.io/tzsgw/.

### Results

A consequence of analyzing Fisher-transformed correlations instead of raw correlations is that the estimator of true effect size becomes slightly underestimated. However, this underestimation is negligible under the selected conditions for sample size and true effect size.^7^ The results of using only the replication data are the reference because the expected value of the replication’s effect size is equal to the population effect size if no *p-*hacking or questionable research practices have been used. Both fixed-effect meta-analysis and the hybrid methods also use the data of the original study. In describing the results, we will focus on answering the question under which conditions these methods will improve upon estimation and testing using only the replication data.

#### Mean and median of effect size estimates

**Fig. 2:**
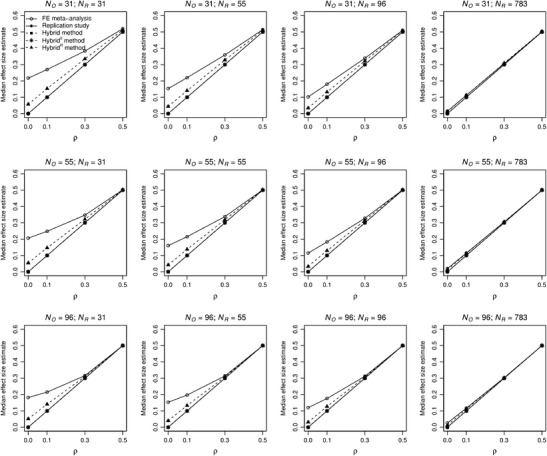
Median effect size estimates of the estimators of fixed-effect meta-analysis (solid line with open bullets), replication study (solid line with filled bullets) and hybrid (dashed line with filled squares), hybrid^0^ (dashed line with asterisks), and hybrid^R^ method (dashed line with filled triangles) as a function of population effect size ρ and sample size of the original study (N_O_) and replication (N_R_). Median effect size estimates of the replication study, hybrid, and hybrid^0^ are exactly equal to the population effect size and therefore coincide

Table 2 shows the methods’ expected values as a function of the population effect size (*ρ*) and sample sizes in the original study (*N*_O_) and the replication (*N*_R_). Expected values of the methods’ estimators at *N*_R_ = 783 are presented in Table 6 of the Appendix because their bias is very small in those conditions. We also present the median effect size estimates (Fig. 2 8), since the expected value of the hybrid method is negative, because hybrid’s estimate becomes highly negative if the conditional probability is close to 1 (in other words, the probability distribution of hybrid’s estimate is skewed to the left). Note that the median effect size estimates of the replication, hybrid, and hybrid^0^ are all exactly equal to each other, and therefore coincide in Fig. 2.

**Table 2:** Effect size estimates and standard deviations of these estimates (in parentheses) for estimators of the fixed-effect meta-analysis, replication study, and hybrid, hybrid^0^, and hybrid^R^ methods, as a function of population effect size *ρ* and the sample size of the original study (*N*_O_) and replication (*N*_R_)

	*ρ*	*N*_R_ = 31			*N*_R_ = 55			*N*_R_ = 96		
		*N*_O_ = 31	*N*_O_ = 55	*N*_O_ = 96	*N*_O_ = 31	*N*_O_ = 55	*N*_O_ = 96	*N*_O_ = 31	*N*_O_ = 55	*N*_O_ = 96
FE	0	0.215 (0.094)	0.207 (0.069)	0.184 (0.049)	0.152 (0.089)	0.16 (0.071)	0.154 (0.053)	0.101 (0.079)	0.115 (0.067)	0.12 (0.053)
	0.1	0.268 (0.093)	0.248 (0.07)	0.217 (0.053)	0.219 (0.088)	0.215 (0.071)	0.198 (0.055)	0.179 (0.078)	0.183 (0.067)	0.177 (0.054)
	0.3	0.381 (0.09)	0.349 (0.076)	0.318 (0.068)	0.357 (0.084)	0.337 (0.072)	0.315 (0.065)	0.338 (0.073)	0.327 (0.065)	0.312 (0.059)
	0.5	0.516 (0.086)	0.499 (0.079)	0.497 (0.068)	0.511 (0.076)	0.499 (0.071)	0.498 (0.062)	0.507 (0.064)	0.5 (0.06)	0.498 (0.055)
Replica-tion	0	0 (0.182)	0 (0.182)	0 (0.182)	0 (0.135)	0 (0.135)	0 (0.135)	0 (0.102)	0 (0.102)	0 (0.102)
	0.1	0.097 (0.18)	0.097 (0.18)	0.097 (0.18)	0.098 (0.134)	0.098 (0.134)	0.098 (0.134)	0.099 (0.101)	0.099 (0.101)	0.099 (0.101)
	0.3	0.291 (0.167)	0.291 (0.167)	0.291 (0.167)	0.295 (0.124)	0.295 (0.124)	0.295 (0.124)	0.297 (0.093)	0.297 (0.093)	0.297 (0.093)
	0.5	0.487 (0.141)	0.487 (0.141)	0.487 (0.141)	0.493 (0.103)	0.493 (0.103)	0.493 (0.103)	0.496 (0.077)	0.496 (0.077)	0.496 (0.077)
Hybrid	0	– 0.013 (0.195)	– 0.016 (0.182)	– 0.019 (0.168)	– 0.007 (0.155)	– 0.01 (0.146)	– 0.012 (0.136)	– 0.004 (0.122)	– 0.006 (0.117)	– 0.007 (0.11)
	0.1	0.083 (0.189)	0.081 (0.173)	0.078 (0.155)	0.09 (0.15)	0.088 (0.139)	0.086 (0.126)	0.094 (0.119)	0.092 (0.112)	0.091 (0.103)
	0.3	0.279 (0.164)	0.28 (0.14)	0.285 (0.112)	0.287 (0.131)	0.287 (0.114)	0.29 (0.094)	0.292 (0.105)	0.292 (0.093)	0.293 (0.079)
	0.5	0.483 (0.123)	0.491 (0.094)	0.496 (0.072)	0.489 (0.099)	0.494 (0.079)	0.497 (0.063)	0.493 (0.08)	0.496 (0.066)	0.498 (0.055)
Hybrid^0^	0	0.072 (0.101)	0.065 (0.09)	0.057 (0.079)	0.058 (0.083)	0.054 (0.075)	0.048 (0.067)	0.047 (0.067)	0.044 (0.062)	0.04 (0.057)
	0.1	0.127 (0.127)	0.12 (0.115)	0.112 (0.102)	0.117 (0.11)	0.112 (0.101)	0.107 (0.092)	0.11 (0.094)	0.107 (0.088)	0.104 (0.081)
	0.3	0.285 (0.149)	0.284 (0.13)	0.287 (0.106)	0.289 (0.126)	0.288 (0.111)	0.29 (0.092)	0.292 (0.103)	0.292 (0.092)	0.293 (0.078)
	0.5	0.483 (0.122)	0.491 (0.093)	0.496 (0.072)	0.489 (0.099)	0.494 (0.079)	0.497 (0.063)	0.493 (0.08)	0.496 (0.066)	0.498 (0.055)
Hybrid^R^	0	0.049 (0.172)	0.043 (0.164)	0.038 (0.157)	0.04 (0.133)	0.036 (0.128)	0.032 (0.122)	0.032 (0.104)	0.03 (0.1)	0.027 (0.096)
	0.1	0.143 (0.164)	0.136 (0.153)	0.128 (0.142)	0.136 (0.128)	0.131 (0.12)	0.125 (0.112)	0.13 (0.1)	0.126 (0.095)	0.122 (0.089)
	0.3	0.323 (0.139)	0.312 (0.123)	0.302 (0.102)	0.321 (0.11)	0.312 (0.099)	0.303 (0.085)	0.319 (0.088)	0.312 (0.08)	0.304 (0.071)
	0.5	0.501 (0.107)	0.495 (0.089)	0.496 (0.072)	0.503 (0.087)	0.497 (0.076)	0.497 (0.063)	0.504 (0.071)	0.498 (0.064)	0.498 (0.055)

The expected values based on the replication are exactly equal to the population effect size for *ρ* = 0 but are slightly smaller than the true value for larger population effect sizes. This underestimation is caused by transforming the Fisher *z* values to correlation coefficients.^9^ The median estimate of the replication is exactly equal to the population effect size in all conditions (solid lines with filled bullets in Fig. 2). Fixed-effect meta-analysis generally yields estimates that are too high when there is no or only a small effect in the population, particularly if the sample sizes are small (bias equal to .215 and .168 for no and small effect). However, its bias is small for a very large sample size in the replication (at most .026, for a zero true effect size and *N*_O_ = 96 and *N*_R_ = 783; see Table 6). Bias decreases as the population effect size and sample size increase, becoming .037 or smaller if the population effect size is at least medium and both sample sizes are at least 55.

The estimator of the hybrid method has a slight negative bias relative to the replication (never more than – 0.021; Table 2) caused by the highly negative estimates if *x* is close to 2 under the null hypothesis. However, its median (dashed lines with filled squares in Fig. 2) is exactly equal to the population effect size. Hybrid^0^, which was developed to correct for the negative bias of hybrid’s estimator, overcorrects and yields an overestimated effect size for *ρ* = 0, with biases equal to .072 and .04 for small and large sample sizes, respectively. The positive bias of hybrid^0^’s estimator is small for a small effect size (at most .027, for small sample sizes), whereas there is a small negative bias for medium and large effect sizes. Hybrid^0^’s median estimate is exactly equal to the population effect size (dashed lines with asterisks in Fig. 2). The results of estimator hybrid^R^ parallel those of hybrid^0^, but with less positive bias for no effect (.049 and .027 for small and large sample sizes, respectively), and more bias for a small effect size (at most .043) and a medium effect size (at most .023). The median estimate of hybrid^R^ (dashed lines with triangles in Fig. 2) slightly exceeds the population effect size, because the data of the original study are omitted only if they indicate a negative effect.

To conclude, the negative bias of the hybrid’s estimator is small, whereas the estimators of hybrid^R^ and hybrid^0^ overcorrect this bias for no and small population effect sizes. The fixed-effect meta-analytic estimator yields severely overestimated effect sizes for no and small population effect sizes, but yields approximately accurate estimates for a large effect size. The bias of all methods decreases if sample sizes increase, and all methods yield accurate effect size estimates for large population effect sizes.

#### Precision

Table 2 also presents the standard deviation of each effect size estimate, reflecting the precision of these estimates. The standard deviations of the effect size estimates for *N*_R_ = 783 are presented in Table 6 and are substantially smaller than the standard deviations of the other conditions for *N*_R_. The fixed-effect meta-analytic estimator yields the most precise estimates. The precision of hybrid’s estimator increases relative to the precision of the replication’s estimator in population effect size and the ratio of original to replication sample size. For zero and small population effect sizes, the estimator of hybrid has lower precision than the replication’s estimator if the replication sample size is equal or lower than the original sample size. For medium and large population effect sizes, the estimator of hybrid generally has higher precision, except when the sample size in the original study is much smaller than the replication’s sample size. The estimators of hybrid^0^ and hybrid^R^ have higher precision than hybrid’s estimator because they deal with the possibly strongly negative estimates of hybrid, with hybrid^0^’s estimator in general being most precise for zero and small population effect sizes, and the estimator of hybrid^R^ being most precise for medium and large population effect sizes. They also have higher precision than the estimator of the replication, but not when the replication’s sample size is larger than the sample size of the original study and at the same time the effect size in the population is medium or large (hybrid^0^; *N*_O_ = 31/55 and *N*_R_ = 96) or zero (hybrid^R^; *N*_O_ = 31 and *N*_R_ = 96).

#### RMSE

**Fig. 3:**
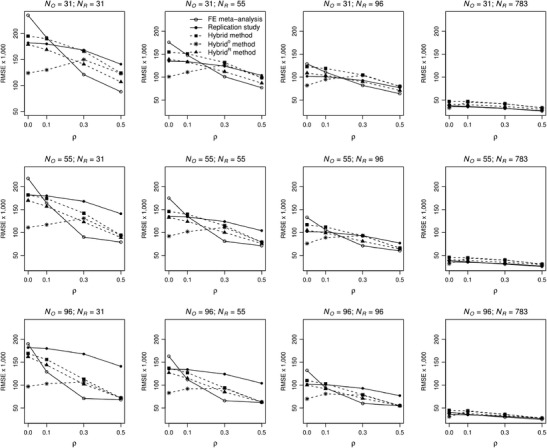
Root mean square errors (RMSE) of the estimators of fixed-effect meta-analysis (solid line with open bullets), replication study (solid line with filled bullets) and hybrid (dashed line with filled squares), hybrid^0^ (dashed line with asterisks), and hybrid^R^ method (dashed line with filled triangles) as a function of population effect size ρ and sample size of the original study (N_O_) and replication (N_R_)

The RMSE combines two important statistical properties of an estimator: bias and precision. A slightly biased and very precise estimator is often preferred over an unbiased but very imprecise estimator. The RMSE is an indicator of this trade-off between bias and precision and is displayed in Fig. 3. As compared to the replication’s estimator, the RMSE of the fixed-effect meta-analytic estimator is higher for no effect in the population, and smaller for medium and large effect sizes. For small population effect sizes, the RMSE of the estimators of the replication and of fixed-effect meta-analysis are roughly the same for equal sample sizes, whereas the RMSE of the replication’s estimator was higher for *N*_O_ > *N*_R_ and lower for *N*_O_ < *N*_R_. Comparing the estimators of hybrid to the replication for equal sample sizes of both studies, hybrid’s RMSE is higher for zero and small population effect sizes, but lower for medium and large population effect sizes. However, the performance of hybrid’s estimator relative to the estimator of the replication depends on both sample sizes and increases with the ratio *N*_O_/*N*_R_. The RMSEs of the estimators of hybrid^0^ and hybrid^R^ are always lower than that of hybrid’s estimator. They are also lower than the RMSE of the replication, except for *N*_O_ = 31 and *N*_R_ = 96 with a zero or small population effect size (hybrid^R^), or a medium or large population effect size (hybrid^0^). The RMSEs of the estimators of hybrid^0^ and hybrid^R^ are lower than that of the fixed-effect meta-analytic estimator for zero or small population effect size, and higher for medium or large population effect size. For *N*_R_ = 783, the RMSEs of all estimators were close to each other (see the figures in the last column of Fig. 3).

#### Statistical properties of the test of no effect

**Fig. 4:**
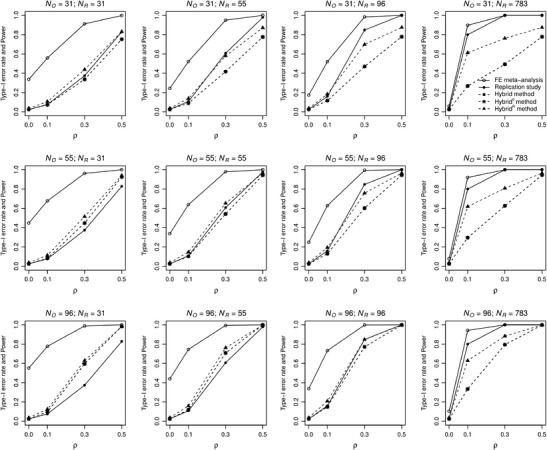
Type I error rate and statistical power of the testing procedures of fixed-effect meta-analysis (solid line with open bullets), replication study (solid line with filled bullets) and hybrid (dashed line with filled squares), hybrid^0^ (dashed line with asterisks), and hybrid^R^ method (dashed line with filled triangles) as a function of population effect size *ρ* and sample size of the original study (*N*_O_) and replication (*N*_R_)

Figure 4 presents the Type I error and statistical power of all methods’ testing procedures. The Type I error rate is exactly .025 for the replication, hybrid, and hybrid^0^ method. The Type I error rate is slightly too high for hybrid^R^ (.037 in all conditions), and substantially too high for fixed-effect meta-analysis (increases with *N*_O_/*N*_R_, up to .551 for *N*_O_ = 96 and *N*_R_ = 31). Concerning statistical power, fixed-effect meta-analysis has by far the highest power, because of its overestimation in combination with high precision. With respect to the statistical power of the other methods, we first consider the cases with equal sample sizes of both studies. Here, hybrid^R^ has highest statistical power, followed by the replication. Hybrid and hybrid^0^ have about equal statistical power relative to the replication for zero and small population effect sizes, but lower statistical power for medium and large population effect sizes. For *N*_O_ > *N*_R_, all hybrid methods have higher power than the replication. For *N*_O_ < *N*_R_ and *N*_R_ < 783, hybrid^R^ has higher statistical power than the replication for zero or small population effect size, but lower statistical power for medium or large population effect size; hybrid and hybrid^0^ have lower statistical power than the replication in this case. The statistical power of the replication is .8 for *ρ* = .1 and *N*_R_ = 783 because the sample size was determined to obtain a power of .8 in this condition, and 1 for *ρ* > .1 and *N*_R_ = 783.

**Fig. 5:**
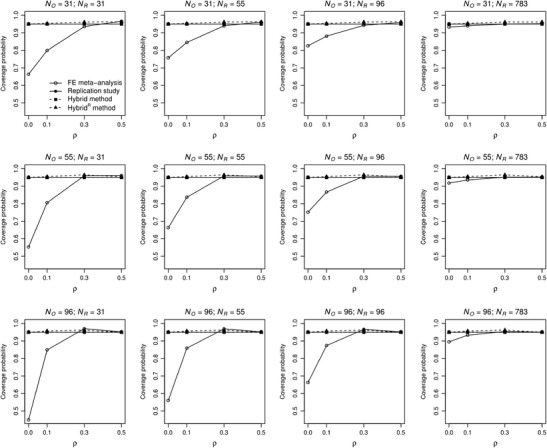
Coverage probabilities of fixed-effect meta-analysis (solid line with open bullets), replication study (solid line with filled bullets) and hybrid (dashed line with filled squares), and hybrid^R^ method (dashed line with filled triangles) as a function of population effect size *ρ* and sample size of the original study (*N*_O_) and replication (*N*_R_)

Coverage is presented in Fig. 5.^10^ The replication and hybrid yield coverage probabilities exactly equal to 95% in all conditions. The coverage probabilities of fixed-effect meta-analysis are substantially too low for *ρ* = 0 and *ρ* = .1, due to overestimation of the average effect size; generally, its coverage improves with effect size and ratio *N*_R_/*N*_O_. The coverage probabilities of hybrid^0^ and hybrid^R^ are close to .95 in all conditions.

#### Guidelines for applying methods

Using the methods’ statistical properties, we attempted to answer the essential question of which method to use under what conditions. Answering this question is difficult because an important condition, population effect size, is unknown, and in fact has to be estimated and tested. We present guidelines (Table 3) that take this uncertainty into account. Each guideline is founded on and explained by using the previously described results.

**Table 3:** Guidelines for applying which method to use when statistically combining an original study and replication

(1a) *When uncertain about population effect size* and sample size in the replication is larger than in the original study (*N*_R_ > *N*_O_), use only the replication data.
(1b) *When uncertain about population effect size* and the sample size in the replication is equal or smaller than in the original study (*N*_R_ ≤ *N*_O_), use hybrid^R^.
(2) *When suspecting zero or small population effect size*, use hybrid^R^
(3) *When suspecting medium or larger population effect size*, use fixed-effect meta-analysis.

The hybrid method and its variants have good statistical properties when testing the hypothesis of no effect—that is, both the Type I error rate and coverage are equal or close to .025 and 95%, respectively. Although the methods show similar performance, we recommend using hybrid^R^ over the hybrid and hybrid^0^ methods. Hybrid^R^’s estimator has a small positive bias, but this bias is less than that of hybrid^0^’s estimator if the population effect size is zero. Moreover, hybrid^R^’s estimator has a lower RMSE than hybrid and has higher power than the testing procedures of hybrid and hybrid^0^. Hence, in the guidelines we consider when to use only the replication, fixed-effect meta-analysis, or hybrid^R^.

If the magnitude of the effect size in the population is uncertain, fixed-effect meta-analysis has to be discarded, because it generally yields a highly overestimated effect size and a too-high Type I error rate when the population effect size is zero or small (Guideline 1, Table 3). If the replication’s sample size is larger than that of the original study, we recommend using only the replication (Guideline 1a), because then the replication outperforms hybrid^R^ with respect to power and provides accurate estimates. Additionally, the RMSE of the replication relative to hybrid^R^ gets more favorable with increasing *N*_R_/*N*_O_.

In the case of uncertainty about the magnitude of the population effect size when the sample size in the replication is smaller than that in the original study, we recommend using hybrid^R^ (Guideline 1b), because the estimator of hybrid^R^ outperforms the replication’s estimator with respect to RMSE, and the testing procedure of hybrid^R^ yields greater statistical power than the procedure of the replication. For this situation, including the original data is beneficial, since they contain sufficient information to improve the estimation of effect size relative to using only the replication data. A drawback of using the hybrid^R^ method is that its Type I error rate is slightly too high (.037 vs. .025), but a slightly smaller α- level can be selected to decrease the probability of falsely concluding that an effect exists. If information on the population effect size is known on the basis of previous research, it is valuable to include this information in the analysis (akin to using an informative prior distribution in Bayesian analyses). If the population effect size is suspected to be zero or small, we also recommend using hybrid^R^ (Guideline 2), because its estimator then has lower RMSE and only a small positive bias, and its testing procedure has higher statistical power than the replication. Fixed-effect meta-analysis should be abandoned in this case because its estimator overestimates zero and small population effects.

Fixed-effect meta-analysis is recommended if a medium or larger population effect size is expected (Guideline 3). Bias of the fixed-effect meta-analytic estimator is minor in this case, but its RMSE is smaller, and the testing procedure has a greater statistical power than of any other method. An important qualification of this guideline is the sample size of the original study, because bias is a decreasing function of *N*_O_. If *N*_O_ is small, the statistical power of the original study’s testing procedure is small when the population effect size is medium, and consequently the original’s effect size estimate is generally too high. Hence, to be on the safe side, if expecting a medium population effect size in combination with a small sample size in the original study, one can decide to use only the replication data (if *N*_R_ > *N*_O_) or hybrid^R^ (if *N*_R_ ≤ *N*_O_). When expecting a large population effect size and the main focus is not only on effect size estimation, but also on testing, fixed-effect meta-analysis is the optimal choice. However, if the ultimate goal of the analysis is to get an unbiased estimate of the effect size, only the replication data should be used for the analysis: The replication is not published, and its effect size estimate is therefore not affected by publication bias. Of course, the replication only provides an unbiased estimate if the research is conducted well—for instance, no questionable research practices were used.

### Reproducibility Project: Psychology

The RPP was initiated to examine the reproducibility of psychological research (Open Science Collaboration, 2015). Articles from three high-impact psychology journals (*Journal of Experimental Psychology: Learning, Memory, and Cognition* [JEP: LMC], *Journal of Personality and Social Psychology* [JPSP], and *Psychological Science* [PSCI]) published in 2008 were selected to be replicated. The key effect of each article’s final study was replicated according to a structured protocol, with the authors of the original study being contacted for study materials and reviewing the planned study protocol and analysis plan to ensure the quality of the replication.

A total of 100 studies were replicated in the RPP. One requirement for inclusion in our analysis was that the correlation coefficient and its standard error could be computed for both the original study and the replication. This was not possible for 27 study pairs.^11^ Moreover, transforming the effect sizes to correlation coefficients may have biased the estimates of the hybrid method, since *q*_O_ and *q*_R_ might not exactly be uniformly distributed at the true effect size due to the transformation. We examined the influence of transforming effect sizes to correlation coefficients on the distributions of *q*_O_ and *q*_R_, and concluded that the transformation of effect size will hardly bias the effect size estimates of the hybrid method (see the supplemental materials).

Another requirement for including a study pair in the analysis was that the original study had to be statistically significant, which was not the case for six studies. Hence, fixed-effect meta-analysis and the hybrid methods could be applied to 67 study pairs. The effect sizes of these study pairs and the results of applying fixed-effect meta-analysis and the hybrid methods are available in Table 7 in the Appendix. For completeness, we present the results of all three hybrid methods. The results in Table 7 show that hybrid^0^ set the effect size to zero in 11 study pairs (16.4%)—that is, where the hybrid’s effect size was negative—and that hybrid^R^ also yielded 11 studies with results different from hybrid (16.4%); in five studies (7.5%), all three hybrid variants yielded different estimates.

Table 4 summarizes the resulting effect size estimates for replication, fixed-effect meta-analysis, and the hybrid methods. For each method, the mean and standard deviation of the estimates and the percentage of statistically significant results (i.e., *p* < .05) are presented. The columns in Table 4 refer to the overall results or to the results grouped per journal. Since PSCI is a multidisciplinary journal, the original studies published in PSCI were classified as belonging to cognitive or social psychology, as in Open Science Collaboration (2015).

**Table 4:** Summary results of effect size estimates and percentages of times the null hypothesis of no effect was rejected of fixed-effect meta-analysis (FE), replication, hybrid, hybrid^R^, and hybrid^0^ methods to 67 studies of the Reproducibility Project: Psychology

		Overall	JEP: LMC	JPSP	PSCI: Cog.	PSCI: Soc.
Number of study pairs		67	20	18	13	16
Mean (*SD*)	FE	0.322 (0.229)	0.416 (0.205)	0.133 (0.083)	0.464 (0.221)	0.300 (0.241)
	Replication	0.199 (0.280)	0.291 (0.264)	0.026 (0.097)	0.289 (0.365)	0.206 (0.292)
	Hybrid	0.250 (0.263)	0.327 (0.287)	0.071 (0.087)	0.388 (0.260)	0.245 (0.275)
	Hybrid^0^	0.266 (0.242)	0.353 (0.237)	0.080 (0.075)	0.400 (0.236)	0.257 (0.259)
	Hybrid^R^	0.268 (0.254)	0.368 (0.241)	0.083 (0.093)	0.394 (0.272)	0.247 (0.271)
%Significant results (i.e., *p* value < .05)	FE	70.1%	90%	44.4%	92.3%	56.2%
	Replication	34.3%	50%	11.1%	46.2%	31.2%
	Hybrid	28.4%	45%	11.1%	30.8%	25%
	Hybrid^0^	28.4%	45%	11.1%	30.8%	25%
	Hybrid^R^	34.3%	55%	16.7%	38.5%	25%

% Significance was based on two-tailed *p* values; JEP: LMC = *Journal of Experimental Psychology: Learning, Memory, and Cognition*; JPSP = *Journal of Personality and Social Psychology*; PSCI: cog. = *Psychological Science* cognitive psychology; PSCI: soc. = *Psychological Science* social psychology

The estimator of fixed-effect meta-analysis yielded the largest average effect size estimate (0.322) and the highest percentage of statistically significant results (70.1%). We learned from the previous section to distrust these high numbers when we are uncertain about the true effect size, particularly in combination with a small sample size in the original study. The estimator of the replication yielded on average the lowest effect size estimates (0.199), with only 34.3% of cases in which the null hypothesis was rejected. The estimators of the hybrid variants yielded a higher average estimate (0.250–0.268), with an equal (hybrid^R^) or a lower (hybrid and hybrid^0^) percentage rejecting the null hypothesis of no effect, relative to simple replication. The lower percentage of rejections of the null hypothesis by the hybrid methods is caused not only by the generally lower effect size estimates, but also by the much higher uncertainty of these estimates. The methods’ uncertainty values, expressed by the average widths of the confidence intervals, were 0.328 (fixed-effect meta-analysis), 0.483 (replication), 0.648 (hybrid), 0.615 (hybrid^0^), and 0.539 (hybrid^R^). The higher uncertainty from the hybrid methods than from the replications demonstrates that controlling for the significance of the original study may come at a high cost (i.e., an increase in uncertainty relative to estimation by the replication only), particularly when the ratio of the replication’s to the original’s sample size gets larger.

If we apply our guidelines to the data of the RPP and suppose that we are uncertain about the population effect size (Guidelines 1a and 1b in Table 3), only the replication data are interpreted in 43 cases, because *N*_R_ > *N*_O_, and hybrid^R^ is applied 24 times (*N*_O_ ≥ *N*_R_). The average effect size estimate of the replication’s estimator with *N*_R_ > *N*_O_ is lower than that of the fixed-effect meta-analytic estimator (0.184 vs. 0.266), and the number of statistically significant pooled effect sizes is also lower (34.9% vs. 55.8%). The average effect size estimate of hybrid^R^’s estimator applied to the subset of 24 studies with *N*_O_ ≥ *N*_R_ is also lower than that of the fixed-effect meta-analytic estimator (0.375 vs. 0.421), and the same holds for the number of statistically significant results (54.2% vs. 95.8%).

The results per journal show higher effect size estimates and more rejections of the null hypothesis of no effect for cognitive psychology (JEP: LMC and PSCI: cog.) than for social psychology (JPSP and PSCI: soc.), independent of the method. The estimator of fixed-effect meta-analysis yielded higher estimates, and the null hypothesis was more often rejected than with the other methods. The estimates of the replication were always lower than those of the hybrid methods. The numbers of statistically significant results of hybrid and hybrid^0^ were equal to or lower than with replication, whereas the number of statistically significant results of hybrid^R^ was equal to or higher than with either hybrid or hybrid^0^. Particularly striking are the low numbers of statistically significant results for JPSP: 16.7% (hybrid^R^) and 11.1% (replication, hybrid, and hybrid^0^).

We also computed a measure of association, to examine how often the methods yielded the same conclusions with respect to the test of no effect, for all study pairs both together and grouped per journal. Since this resulted in a dichotomous variable, we used Loevinger’s *H* (Loevinger, 1948) as the measure of association. Table 5 shows Loevinger’s *H* of the replication as compared to each other method for all 67 study pairs. The associations between fixed-effect meta-analysis, hybrid, hybrid^0^, and hybrid^R^ were perfect (*H* = 1), implying that a hybrid method only rejected the null hypothesis if fixed-effect meta-analysis did as well. The associations of the replication with hybrid, hybrid^0^, and hybrid^R^ were .519, .519, and .603, respectively.

**Table 5:** Loevinger’s *H* across all 67 studies of all methods’ results of hypothesis testing

	FE	Hybrid	Hybrid^0^	Hybrid^R^
Replication	1	.519	.519	.603
FE		1	1	1
Hybrid			1	1
Hybrid^0^				1
Hybrid^R^				

JEP: LMC = *Journal of Experimental Psychology: Learning, Memory, and Cognition*; JPSP = *Journal of Personality and Social Psychology*; PSCI: cog. = *Psychological Science*, cognitive psychology; PSCI: soc. = *Psychological Science*, social psychology

To conclude, when correcting for the statistical significance of the original study, the estimators of the hybrid methods on average provided smaller effect size estimates than did the fixed-effect meta-analytic estimator. The uncertainty of the hybrid estimators (the width of the confidence interval) was invariably larger than that of the fixed-effect meta-analytic estimator, which together with their lower estimates explain the hybrids’ lower percentages of rejections of the null hypothesis of no effect. If a hybrid method rejected the null hypothesis, this hypothesis was also rejected by fixed-effect meta-analysis, but not the other way around. This suggests that the testing procedures of the hybrid methods are primarily more conservative than the testing procedure of fixed-effect meta-analysis. As compared to the replication alone, the hybrid methods’ estimators on average provided somewhat larger effect sizes, but higher uncertainties, with similar percentages reflecting how often the null hypothesis of no effect was rejected. The results of the hybrid methods were more in line with those of only the replication than with the results of fixed-effect meta-analysis or the original study.

## Discussion

One of the pillars of science is replication; does a finding withstand replication in similar circumstances, or can the results of a study generalized across different settings and people, and do the results persist over time? According to Popper (1959/2005), replications are the only way to convince ourselves that an effect really exists and is not a false positive. The replication issue is particularly relevant in psychology, which shows an unrealistically high rate of positive findings (e.g., Fanelli, 2012; Sterling et al., 1995). The RPP (Open Science Collaboration, 2015) replicated 100 studies in psychology and confirmed these unrealistic findings; less than 40% of original findings were statistically significant. The present article examined several methods for estimating and testing effect size combining a statistically significant effect size of the original study and effect size of a replication. By approximating analytically the joint probability density function of original study and replication effect size we show that the estimator of fixed-effect meta-analysis yields overestimated effect size, particularly if the population effect size is zero or small, and yields a too high Type I error rate. We developed a new method, called hybrid, which takes into account that the expected value of the statistically significant original study is larger than the population effect size, and enables point and interval estimation, and hypothesis testing. The statistical properties of hybrid and two variants of hybrid are examined and compared to fixed-effect meta-analysis and to using only replication data. On the basis of this comparison, we formulated guidelines for when to use which method to estimate effect size. All methods were also applied to the data of the RPP.

The hybrid method is based on the statistical principle that the distribution of *p* values at the population effect size has to be uniform. Since positive findings are overrepresented in the literature, the method computes probabilities at the population effects size for both the original study and replication in which likely overestimation of the original study is taken into account. The hybrid method showed good statistical properties (i.e., Type I error rate equal to α- level, coverage probabilities matching the nominal level, and median effect size estimate equal to the population effect size) when its performance was analytically approximated. However, hybrid’s estimator is slightly negatively biased if the mean of the (conditional) probabilities was close to 1. This negative bias was also observed in another meta-analytic method (*p-*uniform) using conditional probabilities. To correct for this bias, we developed two alternative methods (hybrid^0^ and hybrid^R^) that do not suffer from these highly negative estimates and have the same desirable statistical properties as the hybrid method. We recommend using the hybrid^R^ method among the three hybrid variants because its estimator is least biased, its RMSE is lower than hybrid’s estimator, and hybrid^R^’s testing procedure has the most statistical power.

We formulated guidelines (see Table 3) to help researchers select the most appropriate method when combining an original study and replication. The first two guidelines suppose that a researcher does not have knowledge about the magnitude of the population effect size. In this case, we advise to use only the replication data if the original study’s sample size is smaller than of the replication and to use the hybrid^R^ method if the sample size in the original study is larger or equal to the sample size of the replication. The hybrid^R^ method is also recommended to be used if the effect size in the population is expected to be either absent or small. Fixed-effect meta-analysis has the best statistical properties and is advised to be used if the expected population effect size is medium or large. To prevent researchers from selecting a method on the basis of its results (“*p*-hacking”), we recommend selecting the method using our guidelines *before* analyzing the data.

Applying the hybrid methods to studies of RPP largely confirmed the results of only the replication study as reported by the Open Science Collaboration (2015). Average effect size and proportion of statistically significant effects was considerably larger for fixed-effect meta-analysis than for the other methods, providing indirect evidence of overestimation by fixed-effect meta-analysis. The results suggest that many findings published in the three included psychology journals have smaller effect sizes than reported and that some effects may even be absent. In addition, uncertainty of the estimates of the hybrid methods was generally high, meaning that discarding the original studies generally made effect size estimates more precise. We draw two general conclusions from our reanalysis of the RPP. First, estimates of only the replication and the hybrid methods are generally more accurate than both the original study and fixed-effect meta-analysis that tend to overestimate because of publication bias. Second, most estimates of the replication and the hybrid methods were too uncertain to draw strong conclusions on the magnitude of the effect size—that is, sample sizes were too small to provide precise estimates. These two conclusions are in line with a Bayesian re-analysis of the RPP (Etz & Vandekerckhove, 2016).

The effect size estimates of the hybrid methods can also be used to estimate the power of the original study, on the basis of hybrid’s effect size estimate. This alternative calculation of so-called ‘observed power’ has the advantage that it is based on evidence of both the original study and the replication. The observed power of the original study may be interpreted as an index of the statistical quality of the original study, with values of .8 or higher signaling good quality (Cohen, 1990). However, we recommend caution in interpreting this alternative observed value, because it is imprecise particularly when both studies’ sample sizes is low. To work out an example of this approach we applied it to the example in the introduction and Table 1. Following our guidelines in Table 3, we use the replication’s effect size estimate equal to *d* = 0.164 in combination with the original sample size equal to 80 for our power analysis. Entering these numbers in G*Power 3.1.9.2 (Faul, Erdfelder, Lang, & Buchner, 2007) yields a power equal to .18 of a one-tailed *t* test (*α* = .05), suggesting that the original study had low statistical quality.

**Fig. 6:**
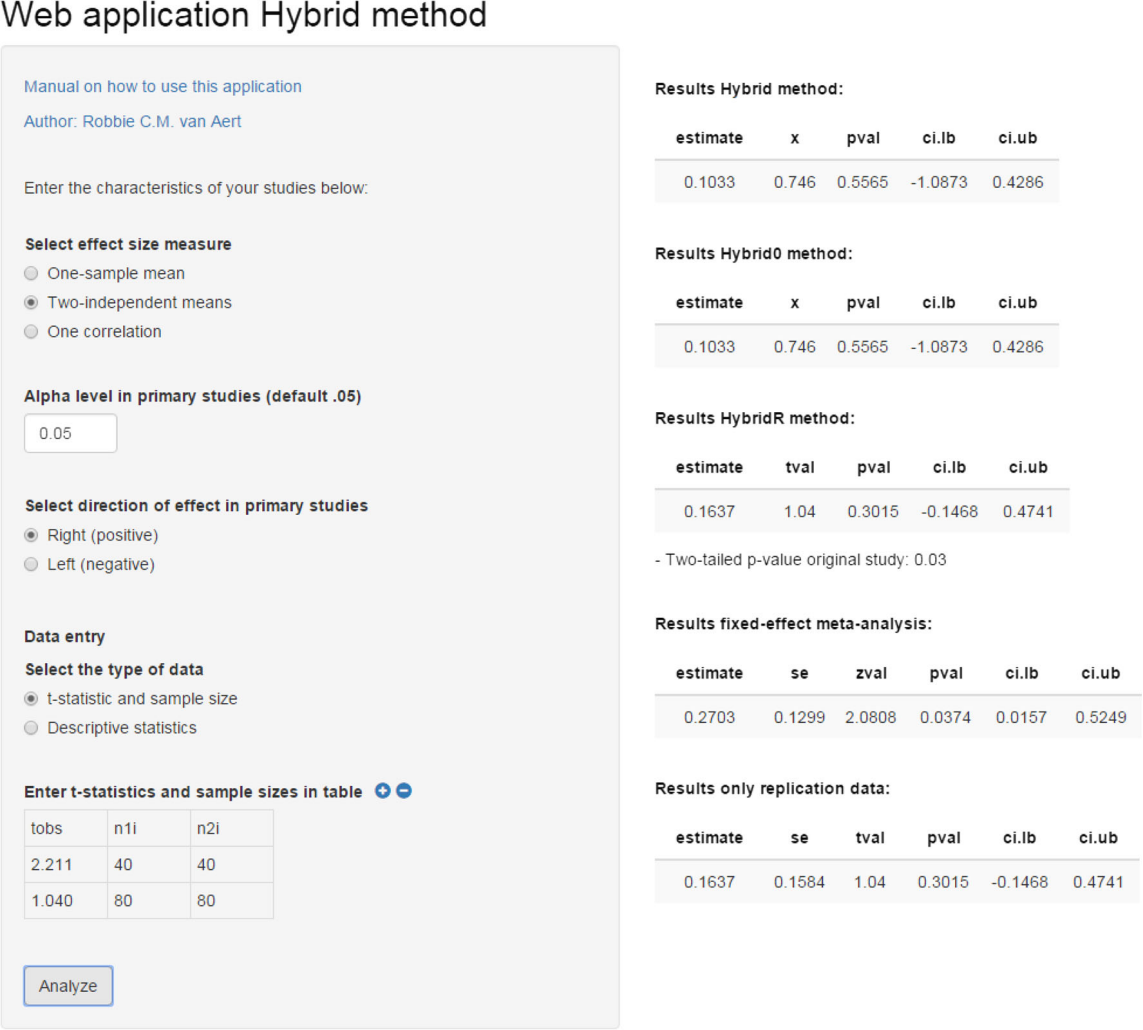
Screenshot of the Web-based application, showing the results of applying the hybrid variants, fixed-effect meta-analysis, and replication to the exemplary data presented in the introduction

We developed R code^12^ and a Web-based application that enables researchers to apply the hybrid methods, as well as fixed-effect meta-analysis, to their own data (https://rvanaert.shinyapps.io/hybrid). Although the hybrid methods can in principle be applied to any effect size measure, the software can currently be applied to three different effect size measures: one-sample mean, two-independent means, and correlation coefficients. For the effect size measures one-sample mean and two-independent means, Hedges’ *g* effect sizes and their sampling variances are computed by the software before the methods are applied. This is the same procedure illustrated when we applied the hybrid method to the example in the introduction. If correlation coefficients are used as the effect size measure (as was the case in the application to the RPP data), the software first transforms the correlation coefficients to Fisher-transformed correlation coefficients and computes the corresponding sampling variances. The Fisher-transformed correlation coefficients and their sampling variances are then used for applying the methods, where the output provides the back-transformed correlation coefficients. Figure 6 shows a screenshot of the application after it was applied to the example presented in the introduction. Data for one-sample mean and two-independent means can be entered via either group means, sample sizes, and standard deviations or *t* values and sample sizes. Users should also specify the α- level and the direction of the hypothesis test that was used in the primary studies. The right-hand side of the Web application presents the results (showing the estimate, test statistic [*t* value, *z* value, or *x*], two-tailed *p* value, and confidence interval) of hybrid, hybrid^0^, hybrid^R^, fixed-effect meta-analysis, and the replication. The application includes a link to a short manual on how to use the application.

The hybrid methods assume that researchers have selected statistically significant original findings to replicate. The expected value of a statistically significant finding exceeds the population effect size, irrespective of publication bias, and the hybrid method corrects for this overestimation. A critical question is how to estimate effect size if a researcher wants to replicate a statistically significant original study, but this study was *not* selected because of its significance. How to proceed in this case depends on the existence of publication bias. If no publication bias exists in the study’s field, fixed-effect meta-analysis is the optimal method to combine an original study and replication, assuming that both estimate the same underlying true effect size. However, if strong publication bias exists, as seems to be the case in psychology, the literature rather than the researcher has already mainly selected the statistically significant findings. Thus, even though researchers did not select a study to replicate on the basis of its being statistically significant, we recommend applying the presented guidelines (Table 3) because the literature mainly presents significant and overestimated effect size estimates.

Another assumption of the hybrid methods is that a common effect (i.e., a fixed effect) underlies the original study and replication. This assumption can be violated if there are substantial discrepancies between the original study and replication. These discrepancies may be caused by differences in the methodologies used in both studies (Gilbert, King, Pettigrew, & Wilson, 2016). Discrepancies may also be caused by findings that can only be replicated under specific conditions and that do not generalize to different settings or subjects, or that do not persist over time (Amir & Sharon, 1990; Henrich, Heine, & Norenzayan, 2010; Klein et al., 2014; S. Schmidt, 2009). Although the assumption of homogeneity in effect sizes can be tested in a meta-analysis, it is difficult to draw reliable inferences in the case of only two studies. The *Q* test, which is used for testing homogeneity, lacks statistical power if the number of studies in a meta-analysis is small (e.g., Borenstein et al., 2009, chap. 16; Jackson, 2006).

We will extend the hybrid methods such that they can include more than one original study and one replication. These extended hybrid methods can be applied if, for instance, a researcher replicates a finding on which multiple original studies or a meta-analysis has already been published. These variants would use only the statistically significant findings of the original studies or meta-analysis, as does *p*-uniform (van Aert et al., 2016, 2015), and would combine these with the replication finding(s) to estimate common effect size.

An important implication of our analysis is that it may be optimal to discard information of the original study when estimating effect size. This is the case when being uncertain about population effect size and sample size in the replication is larger than in the original study, a situation that occurs very frequently. For instance, the sample size of 70 out of 100 replications in RPP is larger in the replication than in the original study. This implication may be generalized when multiple original studies and one replication are combined. Fixed-effect meta-analyses overestimate particularly if they incorporate more original studies with a relatively small sample size, and accuracy of estimation is better served by one or few large studies (Button et al., 2013; Gerber, Green, & Nickerson, 2001; Kraemer, Gardner, Brooks, & Yesavage, 1998; Nuijten, van Assen, Veldkamp, & Wicherts, 2015). We contend that extended hybrid methods, although they can correct for probable overestimation by original studies in the meta-analysis, their accuracy and precision is better served by more replication studies. Discarding all original studies and estimation by only one or a few large replication studies may even be the optimal choice (Nuijten et al., 2015). Omitting biased original studies from a meta-analysis is not a research waste since the effect size estimate will become more accurate.

The present study has several limitations that offer opportunities for future research. First, at present the hybrid method only allows for estimation based on one original and one replication study. We plan to extend the hybrid method to incorporate multiple original and replication studies, and to examine its performance as a function of true effect size, publication bias, and the number of studies and their sample sizes. Second, *p*-hacking or questionable research practices distort the distribution of *p* values, and therefore also of conditional probabilities (Bruns & Ioannidis, 2016; Simonsohn et al., 2014a; Ulrich & Miller, 2015; van Aert et al., 2016, 2015), which will bias the effect size estimates of the hybrid methods. However, note that the results of traditional meta-analytic methods are also distorted by *p*-hacking. Future research may examine to what extent the results of the hybrid methods become biased due to *p-*hacking. A third limitation is that the performance of hybrid methods relative to other methods is dependent on the strength of the population effect, which is the object of the research. The guidelines we propose in Table 3 acknowledge this fact by advising the researcher what to do if the magnitude of the population effect size is uncertain. We must note, however, that the guidelines are formulated in the context of sample sizes presently used in psychological research. The guidelines lose their practical relevance if the sample size of the original study and replication allow for accurate effect size estimation in both studies. For instance, if original and replication sample sizes are 2,000 and 2,050, respectively, it would be naive to discard the original study and only use the replication for interpretation (Guideline 1a, Table 3). In that case, fixed-effect meta-analysis is the recommended method, because overestimation due to publication bias is very small at worst.

The unrealistically high rate of statistically significant results in the published psychological literature suggests that the literature is distorted with false-positive results and overestimated effect sizes. Replication research and statistically combining these replications with the published research via meta-analytic techniques can be used to gather insight into the existence of true effects. However, traditional meta-analytic techniques generally yield overestimated effect sizes. We developed hybrid meta-analytic methods and have demonstrated their good statistical properties. We have also proposed guidelines for conducting meta-analysis by combining the original study and replication and provided a Web application (https://rvanaert.shinyapps.io/hybrid) that estimates and tests the effect sizes of all methods described in this article. Applying the hybrid methods and our guidelines for meta-analyzing an original study and replication will give better insight into psychological phenomena by accurately estimating their effect sizes.

### Author note

The preparation of this article was supported by Grant No. 406-13-050 from the Netherlands Organization for Scientific Research. We thank Hilde Augusteijn, Marjan Bakker, Chris Hartgerink, Michèle Nuijten, Coosje Veldkamp, and Jelte Wicherts for their valuable comments on an earlier draft of this article.

### Electronic supplementary material

ESM 1 (DOCX 1372 kb)

## Appendix

**Table 6:** Effect size estimates and standard deviations of this estimate in brackets for the estimators of fixed-effect meta-analysis, replication study and hybrid, hybrid^0^, and hybrid^R^ method as a function of population effect size *ρ* and sample size of the original study (*N*_O_)

	*ρ*	*N*_R_ = 783		
		*N*_O_ = 31	*N*_O_ = 55	*N*_O_ = 96
FE	0	.015 (.034)	.02 (.033)	.026 (.032)
	.1	.112 (.034)	.115 (.033)	.116 (.032)
	.3	.306 (.031)	.305 (.031)	.302 (.03)
	.5	.501 (.026)	.5 (.026)	.5 (.025)
Replication	0	0 (.036)	0 (.036)	0 (.036)
	.1	.1 (.035)	.1 (.035)	.1 (.035)
	.3	.3 (.032)	.3 (.032)	.3 (.032)
	.5	.5 (.027)	.5 (.027)	.5 (.027)
Hybrid	0	– .001 (.047)	– .001 (.046)	– .001 (.045)
	.1	.099 (.047)	.099 (.045)	.099 (.044)
	.3	.299 (.042)	.299 (.04)	.299 (.036)
	.5	.499 (.033)	.499 (.031)	.499 (.028)
Hybrid^0^	0	.019 (.027)	.018 (.026)	.018 (.025)
	.1	.099 (.046)	.099 (.044)	.099 (.043)
	.3	.299 (.042)	.299 (.04)	.299 (.036)
	.5	.499 (.033)	.499 (.031)	.499 (.028)
Hybrid^R^	0	.013 (.039)	.013 (.039)	.012 (.038)
	.1	.112 (.038)	.112 (.038)	.111 (.036)
	.3	.309 (.035)	.306 (.034)	.303 (.033)
	.5	.503 (.03)	.5 (.03)	.499 (.028)

The sample size of the replication (*N*_R_) is 783

**Table 7:** Data of the Reproducibility Project: Psychology and the results of applying fixed-effect meta-analysis and the hybrid, hybrid^0^, and hybrid^R^ methods to these data

Study	*r*_o_ (*N*_O_) [*p* Value]	*r*_r_ (*N*_R_) [*p* Value]	FE MA (95% CI) [*p* Value]	Hybrid (95% CI)[*p* Value]	Hybrid^R^ (95% CI)[*p* Value]
Roelofs (2008)	.595 (15)[.018]	.148 (30)[.437]	.304 (0; .557) [.0498]	.176 (–.347; .615) [.328]	.176 (–.347; .615) [.328]
Morris and Still (2008)	.611 (25)[.001]	.23 (25)[.273]	.44 (.175; .646) [.002]	.405 (.054; .698) [.024]	.405 (.054; .698) [.024]
Liefooghe, Barrouillet, Vandierendonck, and Camos (2008)	.425 (26)[.03]	–.215 (33)[.231]	.073 (–.194; .33) [.594]	–.208 (–.755; .311) [.275]^0^	–.215 (–.52; .138) [.231]
Storm, Bjork, and Bjork (2008)	.229 (192)[.001]	–.006 (270)[.92]	.093 (.001; .183) [.047]	.077 (–.055; .276) [.322]	.077 (–.055; .276) [.322]
Mitchell, Nash, and Hall (2008)	.461 (33)[.006]	.135 (49)[.358]	.272 (.054; .465) [.015]	.217 (–.04; .534) [.093]	.217 (–.04; .534) [.093]
Berry, Shanks, and Henson (2008)	.595 (25)[.001]	.396 (33)[.022]	.487 (.254; .666)[<.001]	.47 (.218; .687) [.001]	.47 (.218; .687) [.001]
Beaman, Neath, and Surprenant (2008)	.715 (101)[<.001]	.131 (16)[.317]	.668 (.552; .759)[<.001]	.6 (–.078; .751) [.1]	.6 (–.078; .751) [.1]
Dodson, Darragh, and Williams (2008)	.561 (39)[<.001]	–.111 (33)[.543]	.287 (.055; .491) [.016]	.232 (–.245; .641) [.535]	.232 (–.245; .641) [.535]
Ganor-Stern and Tzelgov (2008)	.699 (30)[<.001]	.781 (31)[<.001]	.743 (.6; .84) [<.001]	.743 (.599; .838)[<.001]	.743 (.599; .838) [<.001]
Mirman and Magnuson (2008)	.672 (23)[<.001]	.466 (31) [.007]	.561 (.338; .725)[<.001]	.558 (.318; .755)[<.001]	.558 (.318; .755)[<.001]
J. R. Schmidt and Besner (2008)	.195 (96)[.028]	.247 (243)[<.001]	.233 (.129; .331)[<.001]	.19 (–.373; .304) [.321]	.247 (.125; .361)[<.001]
Oberauer (2008)	.56 (33)[.001]	.402 (21)[.071]	.505 (.266; .685)[<.001]	.482 (.204; .666) [.002]	.482 (.204; .666) [.002]
Sahakyan, Delaney, and Waldum (2008)	.224 (96)[.028]	.019 (108)[.842]	.117 (–.022; .251) [.099]	.004 (–.397; .198) [.96]	.019 (–.17; .208) [.842]
Bassok, Pedigo, and Oskarsson (2008)	.364 (154)[<.001]	.284 (50)[.045]	.345 (.217; .462)[<.001]	.335 (.175; .444) [.001]	.335 (.175; .444) [.001]
Yap, Balota, Tse, and Besner (2008)	.378 (33)[.029]	.38 (72)[.001]	.379 (.199; .534)[<.001]	.294 (–.689; .482) [.345]	.38 (.162; .562) [.001]
Turk-Browne, Isola, Scholl, and Treat (2008)	.738 (9)[.021]	.704 (16)[.002]	.715 (.42; .873) [<.001]	.626 (–.635; .84) [.169]	.626 (–.635; .84) [.169]
White (2008)	.623 (38)[<.001]	.481 (39)[.002]	.555 (.374; .695)[<.001]	.554 (.362; .701)[<.001]	.554 (.362; .701)[<.001]
Farrell (2008)	.517 (41)[<.001]	.316 (41)[.044]	.422 (.221; .588)[<.001]	.408 (.179; .603) [.001]	.408 (.179; .603) [.001]
Pacton and Perruchet (2008)	.714 (22)[<.001]	.682 (22)[<.001]	.698 (.497; .828)[<.001]	.696 (.508; .816)[<.001]	.696 (.508; .816) [<.001]
Makovski, Sussman, and Jiang (2008)	.551 (13)[.0499]	.35 (19)[.144]	.433 (.079; .69) [.018]	–.312 (–1; .505) [.865]^0^	.35 (–.124; .694) [.144]
Payne, Burkley, and Stokes (2008)	.352 (69)[.003]	.15 (178)[.045]	.208 (.084; .325) [.001]	.202 (.067; .419) [.006]	.202 (.067; .419) [.006]
Cox et al. (2008)	.225 (94)[.029]	–.052 (194)[.469]	.039 (–.078; .154) [.517]	–.055 (–.425; .169) [.439]^0^	–.052 (–.192; .089) [.469]
Albarracín et al. (2008)	.378 (36)[.022]	–.03 (88)[.779]	.089 (–.091; .263) [.332]	–.013 (–.373; .36) [.894]^0^	–.013 (–.373; .36) [.894]
Centerbar, Schnall, Clore, and Garvin (2008)	.206 (133)[.017]	.094 (113)[.323]	.155 (.03; .275) [.015]	.092 (–.114; .242) [.258]	.092 (–.114; .242) [.258]
Amodio, Devine, and Harmon-Jones (2008)	.377 (33)[.03]	.077 (75)[.514]	.169 (–.023; .35) [.084]	.04 (–.707; .3) [.728]	.077 (–.153; .298) [.514]
van Dijk, van Kleef, Steinel, and van Beest (2008)	.379 (101)[<.001]	–.042 (40)[.798]	.271 (.109; .419) [.001]	.211 (–.166; .442) [.363]	.211 (–.166; .442) [.363]
Lemay and Clark (2008)	.167 (184)[.023]	.037 (280)[.541]	.089 (–.003; .179) [.057]	.033 (–.183; .163) [.536]	.033 (–.183; .163) [.536]
Ersner-Hershfield, Mikels, Sullivan, and Carstensen (2008)	.22 (110)[.021]	–.005 (222) [.944]	.07 (–.038; .177) [.205]	.008 (–.188; .215) [.894]	.008 (–.188; .215) [.894]
Correll (2008)	.274 (70)[.021]	.074 (147)[.375]	.139 (.005; .268) [.042]	.072 (–.244; .27) [.378]	.072 (–.244; .27) [.378]
Exline, Baumeister, Zell, Kraft, and Witvliet (2008)	.432 (43)[.003]	.012 (133)[.894]	.117 (–.033; .262) [.125]	.111 (–.07; .508) [.266]	.111 (–.07; .508) [.266]
Risen and Gilovich (2008)	.186 (118)[.044]	.003 (224)[.964]	.066 (–.041; .172) [.224]	–.065 (–.979; .077) [.413]^0^	.003 (–.128; .134) [.964]
Stanovich and West (2008)	.222 (375)[<.001]	.073 (177)[.332]	.175 (.093; .255)[<.001]	.16 (.016; .26) [.028]	.16 (.016; .26) [.028]
Blankenship and Wegener (2008)	.208 (259)[.001]	.044 (249)[.485]	.129 (.042; .213) [.004]	.114 (–.007; .25) [.066]	.114 (–.007; .25) [.066]
Shnabel and Nadler (2008)	.268 (92)[.009]	–.102 (139) [.234]	.047 (–.083; .176) [.48]	–.02 (–.186; .309) [.861]^0^	–.02 (–.186; .309) [.861]
Goff, Steele, and Davies (2008)	.396 (53)[.003]	.013 (49)[.929]	.22 (.024; .4) [.028]	.156 (–.114; .468) [.277]	.156 (–.114; .468) [.277]
Murray, Derrick, Leder, and Holmes (2008)	.317 (85)[.003]	–.135 (70)[.266]	.119 (–.041; .273) [.144]	.037 (–.228; .379) [.856]	.037 (–.228; .379) [.856]
McCrea (2008)	.344 (28)[.036]	.29 (61)[.012]	.306 (.101; .487) [.004]	.179 (–.926; .41) [.545]	.29 (.041; .505) [.023]
Purdie-Vaughns, Steele, Davies, Ditlmann, and Crosby (2008)	.378 (75)[.001]	–.037 (1488) [.154]	–.017 (–.066; .033) [.506]	.018 (–.057; .448) [.879]	.018 (–.057; .448) [.879]
Dessalegn and Landau (2008)	.382 (36)[.021]	–.223 (47)[.133]	.043 (–.179; .26) [.707]	–.153 (–.44; .374) [.42]^0^	–.153 (–.44; .374) [.42]
Eitam, Hassin, and Schul (2008)	.222 (86)[.039]	–.105 (158)[.19]	.010 (–.116; .136) [.874]	–.146 (–.889; .039) [.094]^0^	–.105 (–.257; .052) [.19]
Farris, Treat, Viken, and McFall (2008)	.554 (280)[<.001]	.091 (144)[.278]	.418 (.335; .494)[<.001]	.385 (.027; .585) [.019]	.385 (.027; .585) [.019]
Janiszewski and Uy (2008)	.333 (57)[.011]	.226 (118)[.014]	.261 (.116; .395)[.001]	.226 (0; .392) [.0501]	.226 (0; .392) [.0501]
McKinstry, Dale, and Spivey (2008)	.701 (11)[.014]	.75 (11)[.006]	.727 (.407; .888)[<.001]	.666 (–.171; .868) [.079]	.666 (–.171; .868) [.079]
Armor, Massey, and Sackett (2008)	.681 (126)[<.001]	.764 (177)[<.001]	.732 (.675; .78) [<.001]	.728 (.643; .787) [<.001]	.728 (.643; .787) [<.001]
Addis, Wong, and Schacter (2008)	.571 (32)[<.001]	.653 (32)[<.001]	.613 (.428; .749)[<.001]	.61 (.409; .742)[<.001]	.61 (.409; .742)[<.001]
Nurmsoo and Bloom (2008)	.502 (33)[.003]	–.45 (10)[.199]	.341 (.033; .59) [.031]	.068 (–.649; .586) [.903]	.068 (–.649; .586) [.903]
Vul and Pashler (2008)	.288 (174)[<.001]	.323 (141)[<.001]	.303 (.199; .401)[<.001]	.303 (.204; .394) [<.001]	.303 (.204; .394)[<.001]
Masicampo and Baumeister (2008)	.214 (113)[.023]	–.049 (160)[.54]	.061 (–.059; .179) [.322]	–.032 (–.237; .2) [.661]^0^	–.032 (–.237; .2) [.661]
Hajcak and Foti (2008)	.38 (31)[.017]	.25 (43)[.053]	.305 (.077; .503) [.009]	.23 (–.191; .464) [.157]	.23 (–.191; .464) [.157]
Alvarez and Oliva (2008)	.722 (9)[.026]	.923 (18)[<.001]	.887 (.754; .951)[<.001]	.847 (–.865; .948) [.261]	.923 (.801; .971)[<.001]
Lau, Kay, and Spencer (2008)	.384 (36)[.02]	–.034 (70)[.779]	.11 (–.085; .297) [.268]	–.003 (–.309; .384) [.98]^0^	–.003 (–.309; .384) [.98]
Winawer, Huk, and Boroditsky (2008)	.685 (30)[<.001]	.527 (27)[.004]	.617 (.418; .759)[<.001]	.613 (.392; .761) [<.001]	.613 (.392; .761)[<.001]
Nairne, Pandeirada, and Thompson (2008)	.446 (25)[.025]	.423 (39)[.007]	.432 (.202; .617)[<.001]	.338 (–.552; .563) [.245]	.338 (–.552; .563) [.245]
Larsen and McKibban (2008)	.21 (117)[.023]	.5 (236)[<.001]	.413 (.322; .496)[<.001]	.382 (–.223; .537) [.209]	.382 (–.223; .537) [.209]
Vohs and Schooler (2008)	.498 (30)[.004]	.102 (58)[.446]	.244 (.032; .434) [.024]	.209 (–.039; .578) [.098]	.209 (–.039; .578) [.098]
Halevy, Bornstein, and Sagiv (2008)	.769 (78)[<.001]	.653 (38)[<.001]	.736 (.638; .811)[<.001]	.726 (.573; .806) [<.001]	.726 (.573; .806)[<.001]
Janssen, Alario, and Caramazza (2008)	.65 (16)[.005]	.497 (13)[.085]	.588 (.26; .795) [.001]	.529 (.109; .768) [.021]	.529 (.109; .768) [.021]
Bressan and Stranieri (2008)	.189 (196)[.008]	–.03 (261)[.628]	.064 (–.028; .155) [.171]	.023 (–.093; .221) [.715]	.023 (–.093; .221) [.715]
Bressan and Stranieri (2008)	.189 (196)[.008]	.018 (316)[.746]	.084 (–.003; .17) [.058]	.055 (–.048; .221) [.284]	.055 (–.048; .221) [.284]
Forti and Humphreys (2008)	.723 (15)[.002]	.208 (20)[.385]	.463 (.136; .699) [.007]	.424 (0; .804) [.0501]	.424 (0; .804) [.0501]
Schnall, Benton, and Harvey (2008)	.4 (43)[.007]	.003 (126)[.975]	.106 (–.047; .254) [.176]	.078 (–.1; .463) [.403]	.078 (–.1; .463) [.403]
Palmer and Ghose (2008)	.86 (9)[.002]	.12 (9)[.768]	.608 (.139; .854) [.014]	.516 (–.211; .917) [.172]	.516 (–.211; .917) [.172]
Heine, Buchtel, and Norenzayan (2008)	.43 (70)[<.001]	.11 (16)[.69]	.383 (.182; .553)[<.001]	.327 (–.101; .517) [.122]	.327 (–.101; .517) [.122]
Moeller, Robinson, and Zabelina (2008)	.31 (53)[.023]	–.034 (72)[.778]	.114 (–.065; .286) [.21]	–.019 (–.354; .287) [.847]^0^	–.019 (–.354; .287) [.847]
Goschke and Dreisbach (2008)	.375 (40)[.017]	.411 (95)[<.001]	.401 (.247; .535)[<.001]	.358 (–.16; .504) [.11]	.358 (–.16; .504) [.11]
Lobue and DeLoache (2008)	.483 (46)[.001]	.178 (46)[.239]	.34 (.141; .512) [.001]	.317 (.055; .564) [.017]	.317 (.055; .564) [.017]
Estes, Verges, and Barsalou (2008)	.595 (19)[.006]	.254 (23)[.245]	.421 (.122; .65) [.007]	.348 (–.017; .678) [.06]	.348 (–.017; .678) [.06]

The first column lists the article from which a key effect was replicated. The next two columns show the correlation coefficient (*r*_o_ and *r*_r_), sample size (*N*_O_ and *N*_R_), and *p* value from the original study and replication, respectively. The final three columns present the average effect size estimate, 95% confidence interval (CI), and *p* value of fixed-effect meta-analysis (FE MA) and the hybrid and hybrid^R^ method. ^0^ behind the estimates of the hybrid method indicates that the hybrid^0^ method would set the average effect size estimate to zero. All *p* values for the original study (second column) and replication (third column) were two-tailed except for those from the studies by Beaman et al. (2008), Schmidt and Besner (2008), McCrea (2008), and Hajcak and Foti (2008). These studies reported one-tailed *p* values. The *p* values for fixed-effect meta-analysis (FE MA), the hybrid and hybrid^R^ methods were two-tailed

## Footnotes

1 Hedges’ *g* is an effect size measure for a two-independent-groups design that corrects for the small positive bias in Cohen’s *d* by multiplying the Cohen’s *d* effect sizes with the correction factor $J=1-\frac{1}{4 df-1}$, where *df* refers to the degrees of freedom (Hedges, 1981). Note that different estimators for effect size in a two-independent-groups design exist, and that Hedges’ *g* and Cohen’s *d* are just two of these estimators (for others, see Viechtbauer, 2007, and Hedges, 1981).

2 Without loss of generality we assume the original study’s effect size is positive. If the original effect size is negative, the direction of the original study, the replication, and the resulting combined estimated effect size should be reversed to obtain the required results.

3 Estimation was based on the Irwin–Hall distribution instead of maximum likelihood. The distribution of the likelihood is typically highly skewed if the true effect size is close to zero and the sample size of the original study is small (as is currently common in psychology), making the asymptotic standard errors of maximum likelihood inaccurate. The probability density function and the cumulative distribution function of the Irwin–Hall distribution are available through the software package Mathematica (Wolfram Research Inc., 2015).

4 The probabilities *q*_O_ and *q*_R_ are not exactly equal to .6 and .15, due to transforming the effect sizes from Cohen’s *d* to Hedges’ *g*. The conditional probabilities based on the transformed effect sizes are ${q}_O=\frac{0.0156}{0.0261}=0.596$ and *q*_R_ = .151. Transforming the effect sizes from Cohen’s *d* to Hedges’ *g* may bias effect size estimates of the hybrid method. We studied to what extent *q*_O_ and *q*_R_ are influenced by this transformation of effect size. This distributions of *q*_O_ and *q*_R_ based on the transformed effect sizes were analytically approximated by means of numerical integration (see the supplementary material for more information and the results), and these distributions should closely follow a uniform distribution according to the theory underlying the hybrid method. The results show that distributions of *q*_O_ and *q*_R_ after the transformation are accurate approximations of uniform distributions. Hence, the transformation from Cohen’s *d* to Hedges’ *g* will hardly bias the estimates of the hybrid method.

5 In case of a two-tailed hypothesis test, the α- level has to be divided by 2 because it is assumed that all observed effect sizes are statistically significant in the same direction.

6 The variance of 1,000 equally spaced probabilities (.08325), which were used to generate the observed effect sizes in the replication, was not exactly equal to the variance in the population (.08333). To examine whether this smaller variance would bias the effect size estimates of the methods, we also computed the effect size estimates for 5,000 equally spaced probabilities for both the original study and replication (i.e., based on 25 instead of 1 million points). These effect size estimates were almost equal to the estimates based on 1,000 equally spaced probabilities (i.e., difference less than .0002). Therefore, we continued using 1,000 equally spaced probabilities for both marginal densities in our analyses.

7 We examined the underestimation caused by transforming the correlations to Fisher-transformed correlations by computing the expected value and variance of the exact probability density distribution of the correlation (Hotelling, 1953) and the probability density distribution of the correlation that is obtained by applying the Fisher transformation. This procedure for computing the expected value and variance is analogous to the one described in Schulze (2004, pp. 119–123). Of the conditions for sample size and true effect size (*ρ*) included in our study, bias in expected value and variance is largest for a sample size of 31 and true effect size of *ρ* = .5. For this condition, the expected value and variance of the exact probability density distribution are .494 and .0260, respectively, and .487 and .0200 for the probability density distribution after applying the Fisher transformation. In other conditions, bias was less than .004 and .002 for the expected value and variance, respectively.

8 A line for each method is drawn through the points in Figs. 2–5 to improve their interpretability. The lines do not reflect extrapolated estimates of the performance of the different methods for true effect sizes that were not included in our analytical approximation.

9 The observed effect sizes were first transformed from Fisher *z* values to correlation coefficients before the average effect size was calculated. This caused a slight underestimation in the effect size estimate based on the replication study.

10 The hybrid^0^ method is omitted from Fig. 5, illustrating the coverage probabilities, because the average effect size estimate was set to zero if the *p* value of the original study was larger than .0125. This made the confidence interval meaningless, since the average effect size estimate could not be included in the confidence interval.

11 If the test statistics of the original study or replication were, for instance, *F*(*df*_1_ > 1, *df*_2_) or *χ* ^2^, the standard error of the correlation coefficient using the Fisher transformation could not be computed, and fixed-effect meta-analysis and the hybrid methods could not be applied to these study pairs.

12 An R function (called *hybrid*) for applying the different hybrid methods is included in the “puniform” package and can be installed by running the following code: devtools::install_github(“RobbievanAert/puniform”).
