# Supplementary material for: Examining reproducibility in psychology: A hybrid method for combining a statistically significant original study and a replication
Source: Behav Res Methods. 2017 Sep 21;50(4):1515–39. doi: 10.3758/s13428-017-0967-6 (PMC6096648; doi:10.3758/s13428-017-0967-6)
Supplement: Supplementary file 1 — (DOCX 1372 kb) [file 13428_2017_967_MOESM1_ESM.docx]

**Supplementary materials**

Examining reproducibility in psychology: A hybrid method for combining a statistically significant original study and a replication

Robbie C. M. van Aert and Marcel A. L. M. van Assen

**The effect of transforming effect sizes on the distribution of *q_O_* and *q_R_***

The hybrid method is based on the statistical principle that *q_O_* and *q_R_* follow a uniform distribution at the true effect size. The transformation of effect size from, for instance, Cohen’s *d* to Hedges’ *g* or from Cohen’s *d* to the correlation coefficient may cause that *q_O_* and *q_R_* do not follow a uniform distribution at the true effect size anymore which will bias the estimates of the hybrid method. Note also that a *t-*distribution is used for computing *q_O_* and *q_R_* when the effect size is Cohen’s *d* whereas a normal distribution is used after transforming effect sizes from Cohen’s *d* to Hedges’ *g.* We studied the impact of effect size transformations on the distribution of *q_O_* and *q_R_*. Effect sizes were transformed from a *t*-distribution (which yields exactly uniform distributions) to distributions based on Hedges’ *g* and the Fisher transformed correlation coefficient which are used for meta-analyses and the hybrid methods. Ideally, the distributions of *q_O_* and *q_R_* are well-approximated by a uniform distribution after both transformations implying that the transformations will not bias effect size estimation.

**Method**

Sample sizes in both groups were set equal to 50, and two different levels of true effect size (δ=0 and 0.2) were selected. One-tailed hypothesis tests with α=.025 were used to assess statistical significance. This corresponds to common practice in psychological research where two-tailed hypothesis tests are conducted and only results in the predicted direction get published. R code of the analyses is available via <https://osf.io/umer9/>.
 The procedure for generating data was analogous to the procedure used for approximating the marginal pdf of the replication as described in the Method section of the paper. Cohen’s *d* effect sizes based on a two-independent groups design were generated by transforming 1,000,000 equally spaced cumulative probabilities to *t*-values and then to Cohen’s *d* effect sizes. The statistics *q_O_* and *q_R_* were computed with Equations (2) and (3) as presented in the paper with the exception that a *t*-distribution was used instead of a normal distribution. The parameter *θ* as defined in Equations (2) and (3) reflects the true effect size and is equal to δ.

Cohen’s *d* effect sizes were subsequently transformed to Hedges’ *g* by multiplying the Cohen’s *d* effect sizes with $J=\frac{\Gamma\left(\frac{df}{2}\right)}{\sqrt{\frac{df}{2}}\Gamma\left(\frac{df-1}{2}\right)}$. Sampling variances of Hedges’ *g* were computed with $\frac{1}{n_{1}}+\frac{1}{n_{2}}+\left[\frac{1-(n_{1}+n_{2}-4)}{(n_{1}+n_{2}-2)J^{2}}\right]g^{2}$ where *n*_1_ and *n*_2_ are the sample sizes for group 1 and 2 and *g* is the Hedges’ *g* effect size. The statistics *q_O_* and *q_R_* were computed with Equations (2) and (3) as presented in the paper with *θ* as defined in those equations being equal to the true effect size δ.
 We subsequently also transformed Cohen’s *d* effect sizes to correlation coefficients using the equations in section 12.5.4 of Borenstein (2009).[[1]](#footnote-1) To resemble the procedure used for analyzing the RPP data, correlation coefficients were transformed by using Fisher’s *r*-to-*z* transformation and their sampling variances were computed with $\frac{1}{n-3}$. Subsequently, *q_O_* and *q_R_* were computed with Equations (2) and (3) as presented in the paper. The parameter *θ*, as defined in Equations (2) and (3) of the paper, was now equal to δ transformed to Fisher transformed correlation coefficient.

**Results** Figures S1 and S2 show the distributions of *q_O_* and *q_R_* for the transformations of effect size as explained above. Figure S1 presents the distributions *q_O_* and *q_R_* for a true effect size equal to δ=0 for Cohen’s *d* (first row of figures) and the transformation to Hedges’ *g* (second row of figures), and δ=0 transformed to Fisher transformed correlation coefficient (third row of figures). Figure S2 entails the same information but then for δ=0.2. Table S1 shows the mean and standard deviation of *q_O_* and *q_R_* for Cohen’s *d* and the transformations of effect size as well as the minimum and maximum density of the distributions in Figure S1 and S2 (i.e., the minimum and maximum height of the bars when a histogram is drawn with 50 bars).

*Table S1.* Mean and standard deviation (SD) of *q_O_* and *q_R_* if Cohen’s *d* was the effect size measure and Cohen’s *d* was transformed to Hedges’ *g* or Fisher transformed correlation coefficient. The last two columns denote the minimal and maximal density when a histogram was drawn with 50 bars. The first and second panel of the table shows the results of δ=0 and δ=0.2, respectively.

|  |  | δ=0 | | |
| --- | --- | --- | --- | --- |
|  |  | Mean (SD) | Min. density | Max. density |
| Cohen’s *d* | *q_O_* | 0.5 (0.289) | 1 | 1 |
|  | *q_R_* | 0.5 (0.289) | 1 | 1 |
| Hedges’ *g* | *q_O_* | 0.502 (0.288) | 0.957 | 1.01 |
|  | *q_R_* | 0.5 (0.288) | 0.974 | 1.005 |
| Fisher correlation | *q_O_* | 0.502 (0.289) | 0.989 | 1.01 |
|  | *q_R_* | 0.5 (0.287) | 0.956 | 1.013 |
|  |  |  |  |  |
|  |  | δ=0.2 | | |
|  |  | Mean (SD) | Min. density | Max. density |
| Cohen’s *d* | *q_O_* | 0.5 (0.289) | 1 | 1 |
|  | *q_R_* | 0.5 (0.289) | 1 | 1 |
| Hedges’ *g* | *q_O_* | 0.505 (0.288) | 0.916 | 1.021 |
|  | *q_R_* | 0.501 (0.288) | 0.961 | 1.006 |
| Fisher correlation | *q_O_* | 0.504 (0.288) | 0.958 | 1.018 |
|  | *q_R_* | 0.499 (0.286) | 0.938 | 1.015 |

The first row of Figure S1 and S2 illustrate the distributions of *q_O_* and *q_R_* if Cohen’s *d* was the effect size measure. The statistics *q_O_* and *q_R_* computed with Cohen’s *d* as effect size measure exactly followed a uniform distribution since these effect sizes were not transformed to another effect size measure. This is also shown in Table S1, because the mean of *q_O_* and *q_R_* for Cohen’s *d* as effect size measure was exactly equal to 0.5, and the minimum and maximum density were both equal to 1. The second row of Figures S1 and S2 present the distributions of *q_O_* and *q_R_* when effect sizes were transformed to Hedges’ *g*. The mean *q_R_* (0.5 for δ=0 and 0.501 for δ=0.2) and minimum (0.961 and 0.974) and maximum (1.005 and 1.006) densities of *q_R_* were very close to their anticipated values whereas these values differed more (means of 0.502 and 0.505, minimum of 0.957 and 0.916, maximum of 1.01 and 1.021), but were still close for *q_O_* (Table S1). As *q_O_*’s density is slightly increasing in *q_O_*, a slight bias in estimation may occur. We verified this by determining the value of true effect size for which the average of *q_O_* exactly equals 0.5 for effect sizes equal to δ=0 and δ=0.2. These values were -0.0004 and 0.191, respectively, corresponding to biases equal to 0.0004 and 0.009. Since these values are very close to the true values of δ=0 and δ=0.2, we conclude that there is hardly any systematic bias in estimation as a consequence of transforming the effect size from Cohen’s *d* to Hedges’ *g*.
 The effects of transforming effect size were also small when transforming Cohen’s *d* effect sizes to Fisher transformed correlation coefficients (last rows of Figure S1 and Figure S2, and Table S1). The density of *q_O_* was only slightly increasing in *q_O_* for δ=0.2, but this hardly resulted in bias as an effect size equal to 0.193 yields an average *q_O_* equal to 0.5 (bias equal to 0.007).

**Conclusions**

Transforming effect sizes from Cohen’s *d* to Hedges’ *g* or from Cohen’s *d* to Fisher transformed correlation coefficients hardly effects the distribution of *q_O_* and *q_R_*. Since the distributions of *q_O_* and *q_R_* are very closely approximated by a uniform distribution, these small deviations hardly bias effect size estimation of the hybrid method. This study examined the effect of transformations in two limited conditions. More research is needed to examine the effect of transformations in other conditions, and on the robustness of the statistical properties of the hybrid methods to violations of other assumptions.


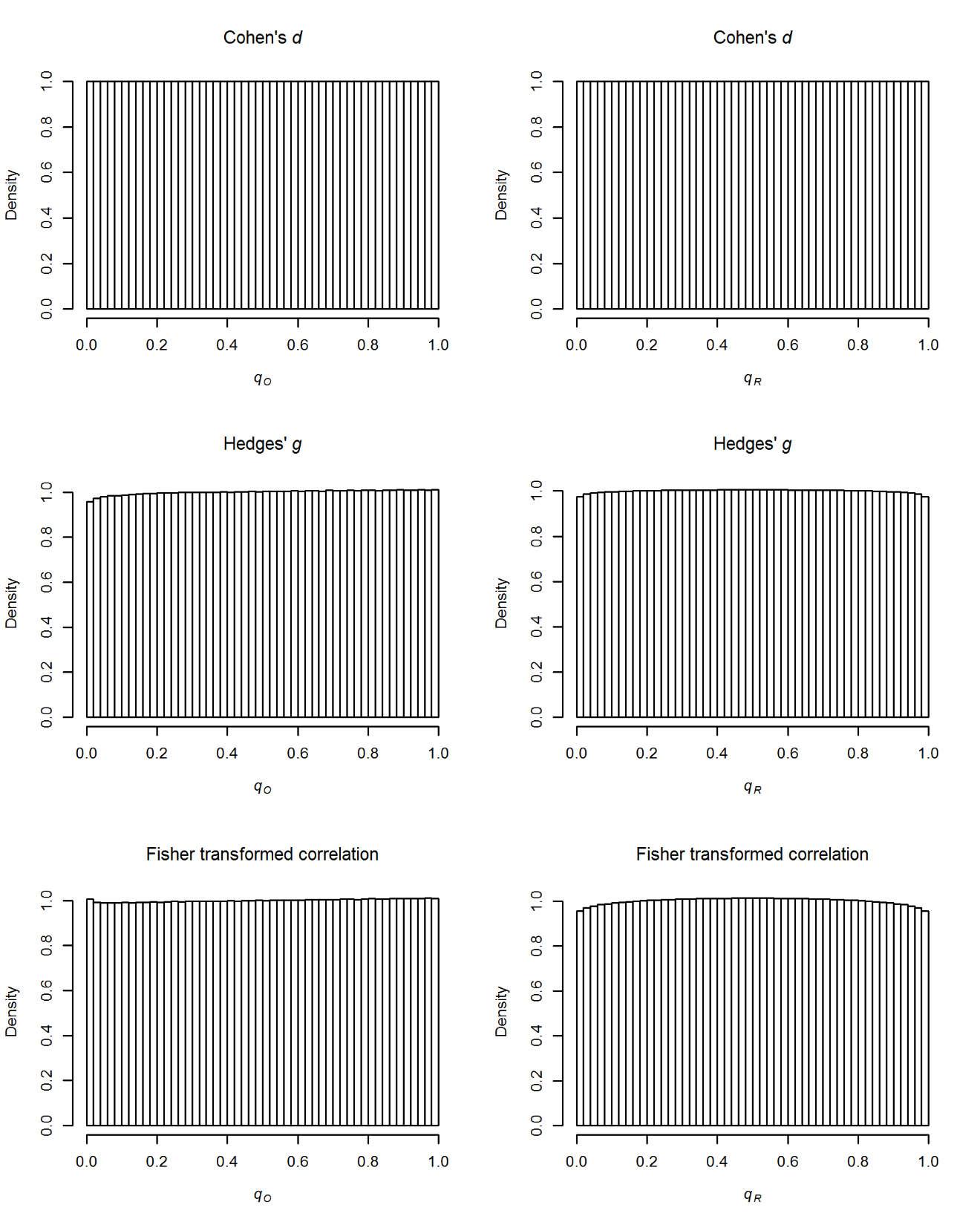


*Figure S1.* Distributions of *q_O_* (first column) and *q_R_* (second column) at the true effect size of δ=0 when the effect size measure is Cohen’s *d* (first row), transformed from Cohen’s *d* to Hedges’ *g* (second row), and transformed from Cohen’s *d* to Fisher transformed correlation coefficient (third row).


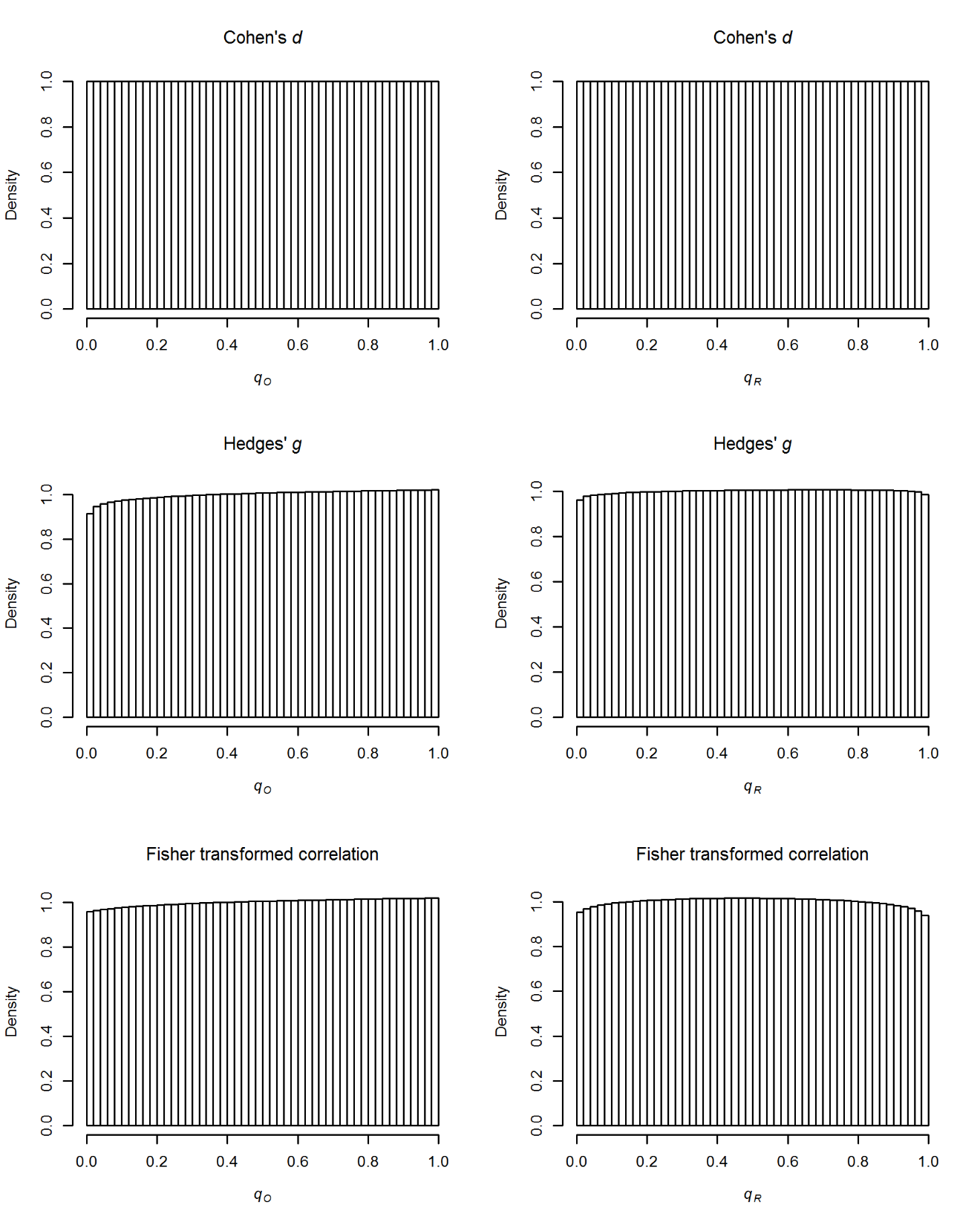


*Figure S2.* Distributions of *q_O_* (first column) and *q_R_* (second column) at the true effect size of δ=0.2 when the effect size measure is Cohen’s *d* (first row), transformed from Cohen’s *d* to Hedges’ *g* (second row), and transformed from Cohen’s *d* to Fisher transformed correlation coefficient (third row).

**References**

Borenstein, M. (2009). Effect sizes for continuous data. In H. Cooper, L. V. Hedges, & J. C. Valentine (Eds.), *The Handbook of Research Synthesis and Meta-Analysis* (pp. 221-236). New York: Russell Sage Foundation.

1. Transforming Cohen’s *d* effect size to correlation coefficient using the formulas in section 12.5.4 of Borenstein (2009) does not result in a Pearson product-moment correlation coefficient but in a point-biserial correlation coefficient. [↑](#footnote-ref-1)
